# A multi-omics study to characterize the transdifferentiation of human dermal fibroblasts to osteoblast-like cells

**DOI:** 10.3389/fmolb.2022.1032026

**Published:** 2022-11-17

**Authors:** Sandra Pihlström, Kirsi Määttä, Tiina Öhman, Riikka E. Mäkitie, Mira Aronen, Markku Varjosalo, Outi Mäkitie, Minna Pekkinen

**Affiliations:** ^1^ Institute of Genetics, Folkhälsan Research Center, Helsinki, Finland; ^2^ Research Program for Clinical and Molecular Metabolism, Faculty of Medicine, University of Helsinki, Helsinki, Finland; ^3^ Institute of Biotechnology and Helsinki Institute of Life Science, University of Helsinki, Helsinki, Finland; ^4^ Department of Otorhinolaryngology—Head and Neck Surgery, Helsinki University Hospital and University of Helsinki, Helsinki, Finland; ^5^ Children’s Hospital, Helsinki University Hospital and University of Helsinki, Helsinki, Finland; ^6^ Department of Molecular Medicine and Surgery and Center for Molecular Medicine, Karolinska Institutet, Stockholm, Sweden

**Keywords:** in vitro method, fibroblasts, transdifferentiation, osteoblast-like cells, osteoblastic differentiation treatment

## Abstract

**Background:** Various skeletal disorders display defects in osteoblast development and function. An *in vitro* model can help to understand underlying disease mechanisms. Currently, access to appropriate starting material for *in vitro* osteoblastic studies is limited. Native osteoblasts and their progenitors, the bone marrow mesenchymal stem cells, (MSCs) are problematic to isolate from affected patients and challenging to expand *in vitro*. Human dermal fibroblasts *in vitro* are a promising substitute source of cells.

**Method:** We developed an *in vitro* culturing technique to transdifferentiate fibroblasts into osteoblast-like cells. We obtained human fibroblasts from forearm skin biopsy and differentiated them into osteoblast-like cells with *ß*-glycerophosphate, ascorbic acid, and dexamethasone treatment. Osteoblastic phenotype was confirmed by staining for alkaline phosphatase (ALP), calcium and phosphate deposits (Alizarin Red, Von Kossa) and by a multi-omics approach (transcriptomic, proteomic, and phosphoproteomic analyses).

**Result:** After 14 days of treatment, both fibroblasts and MSCs (reference cells) stained positive for ALP together with a significant increase in bone specific ALP (*p* = 0.04 and 0.004, respectively) compared to untreated cells. At a later time point, both cell types deposited minerals, indicating mineralization. In addition, fibroblasts and MSCs showed elevated expression of several osteogenic genes (e.g. *ALPL, RUNX2, BMPs* and *SMADs*), and decreased expression of *SOX9*. Ingenuity Pathways Analysis of RNA sequencing data from fibroblasts and MSCs showed that the osteoarthritis pathway was activated in both cell types (p_adj. = 0.003 and 0.004, respectively).

**Discussion:** These data indicate that our *in vitro* treatment induces osteoblast-like differentiation in fibroblasts and MSCs, producing an *in vitro* osteoblastic cell system. This culturing system provides an alternative tool for bone biology research and skeletal tissue engineering.

## Graphical Abstract

**Figure:**
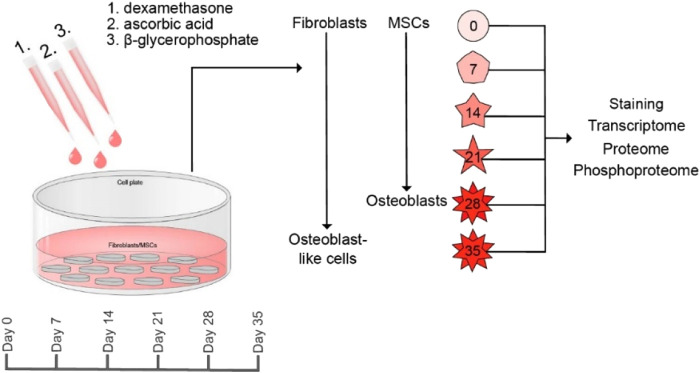
In this study, we developed an *in vitro* technique to transdifferentiate human dermal fibroblasts into osteoblast-like cells. Bone marrow-derived mesenchymal stem cells, the true progenitors of osteoblasts, were utilized as reference cell lines. We characterized the osteoblastic differentiation in the two cell types using transcriptomics and quantitative MS-based proteomics and phosphoproteomics. The osteoblastic-like phenotype was confirmed in both cell types by positive alkaline phosphatase and mineralization staining methods. In addition, both cell types showed altered gene and protein profiles in compliance with an osteoblastic differentiation.

## Introduction

Worldwide, over 650 million fractures occur annually, causing suffering and disability in affected subjects (Wu et al., 2021). The underlying bone pathology is complex and may relate to genetic causes, chronic illnesses, hormonal deficiencies, or medications (Mäkitie and Zillikens, 2021). Several monogenic skeletal diseases, collectively called skeletal dysplasia, present with fractures and other skeletal abnormalities. More than 450 forms have been identified, and in many of them, the genetic cause remains unknown (Mortier et al., 2019).

Several bone disorders are characterized by dysfunction in the commitment, differentiation, survival or function of the osteoblast lineage cells (Marie, 2015). The complex process of bone formation starts with recruitment of bone marrow mesenchymal stem cells (MSCs) followed by cell lineage commitment, osteoblastic differentiation and matrix mineralization before the osteoblasts either die, become flattened lining cells, or are embedded in the bone matrix as osteocytes (Marie, 2015; Rutkovskiy et al., 2016). MSCs can give rise to several lineages. For osteoblastic commitment, the master regulators runt-related transcription factor 2 (RUNX2) and SRY-box transcription factor 9 (SOX9), are essential. In osteochondral progenitors, a critical interplay takes place, where a decrease in *SOX9* expression leads to an increase *RUNX2/SOX9* ratio (Loebel et al., 2015). Once RUNX2 is activated, the cells are defined as preosteoblasts. These cells continue to proliferate and simultaneously express various osteogenic genes, such as genes for collagen, osterix (*SP7*) and osteopontin (*SPP1*), needed for differentiation (Karsenty, 2001). The cells subsequently exit the cell cycle and start to differentiate into osteoblasts (Rutkovskiy et al., 2016). Finally, activating transcription factor 4 (ATF4) is required for terminal differentiation and regulation of bone-forming activities in mature osteoblasts. ATF4 promotes amino acid uptake to facilitate protein synthesis and bone matrix production of osteoblasts (Yang and Karsenty, 2004). Osteoblasts produce and secrete various extracellular proteins, such as alkaline phosphatase (ALP) and osteocalcin (BGLAP). These proteins serve as markers of distinct stages of osteoblastic differentiation. Mature osteoblasts are responsible for bone matrix deposition and mineralization (Rutkovskiy et al., 2016).

Since several skeletal disorders exhibit osteoblast abnormalities, there is a demand for an osteoblastic *in vitro* system to enable detailed functional studies. However, native osteoblasts are difficult to obtain from affected patients and problematic to expand *in vitro* (Notingher et al., 2004). Other cell sources for osteoblastic differentiation *in vitro* are, for instance, bone marrow MSCs and iPSCs (induced pluripotent stem cells) (Jaiswal et al., 1997; Alm et al., 2012; Zhu et al., 2019). However, there are challenges regarding the use of these cells for osteogenic studies. Bone marrow harvesting process is invasive and may trigger bleeding, infections, and discomfort at biopsy site (Bain, 2005). In addition, MSCs are sensitive cells and their *in vitro* culturing requires a close attendance. Similar issues concern the iPSCs. Furthermore, iPSCs can accumulate chromosomal abnormalities, genetic instability, copy number variants and loss of heterozygosity during *in vitro* culturing (Doss and Sachinidis, 2019).

Human dermal fibroblasts are another potential source of cells that are non-immunogenic, easily expandable, and readily available through a minimal invasive harvesting procedure (Hee and Nicoll, 2006). Therefore, we developed an *in vitro* culturing technique to transdifferentiate human fibroblasts into osteoblast-like cells. While previous studies have suggested that such an approach is possible (Hee and Nicoll, 2006), no detailed characteristics or confirmation of the osteogenic nature of the resulting cells have been provided. Therefore, we investigated thoroughly the osteoblast-like cell characteristics in this multi-omics study.

In contrast, methods to differentiate MSCs into fully mature and functional osteoblasts have been well established. This *in vitro* differentiation method is based on adding organic phosphate and ascorbic acid to the cells at an appropriate concentration and timing (Jaiswal et al., 1997; Alm et al., 2012). In addition, dexamethasone is used to induce osteogenesis and mineralization of MSCs. A prolonged exposure to dexamethasone negatively affects mature osteoblasts but a dexamethasone treatment during the first 7 days of the osteoblastic induction of MSC will induce osteogenesis while overcoming the negative effect on osteoblasts (Cheng et al., 1994; Alm et al., 2012).

The hypothesis for the present study was that osteoblastic differentiation treatment of human dermal fibroblasts induces transdifferentiation to osteoblast-like cells. We successfully transdifferentiated human dermal fibroblasts into osteoblast-like cells by treating the cells with *ß*-glycerophosphate, ascorbic acid, and dexamethasone. As a positive reference cell line, we used human commercial MSCs that were differentiated into osteoblasts. The osteoblastic phenotype was verified by staining for ALP and calcium and phosphate deposits (Alizarin Red and Von Kossa staining), and by measuring messenger RNA (mRNA) and protein expression of specific osteoblastic markers. mRNA and protein expression were analyzed by RNA sequencing (RNA-seq) and proteomic analysis. RNA-seq findings were further validated by quantitative reverse transcription polymerase chain reaction (qRT-PCR). This technique provides a novel and usable approach to bone biology research and skeletal tissue engineering.

## Materials and methods

### Study permits and collection of skin biopsies

The study was approved by the ethics committee of the Helsinki University Hospital (HUS/26/2018), and all participants signed an informed consent. All data have been analyzed and stored according to the General Data Protection Regulation (GDPR, EU). Skin biopsies were collected from four healthy individuals participating in the bone metabolism study: fibroblast 1 (male, age 28 years), fibroblast 2 (female, 38 years), fibroblast 3 (female, 50 years) and fibroblast 4 (male, 24 years).

### Fibroblast isolation and culture

After skin biopsy, the biopsies were processed into primary fibroblast cells as previously described (Vakkilainen et al., 2019). Fibroblasts (from passage 4) were cultured in Dulbecco’s Modified Eagle´s Medium (DMEM) (12,492,013, Gibco, Waltham, MA, United States) containing 10% fetal bovine serum (FBS) (S-FEB-SA-015, Serana, Brandenburg, Germany*), 100 IU/ml penicillin, 100 µg streptomycin (15,140,122, Gibco, Waltham, MA, United States) and 2 mM glutamax (35050-038, Gibco, Waltham, MA, United States). The commercial human bone marrow-derived MSCs, named MSC1, MSC2 and MCS3, were purchased from Gibco (A15652, Lot No: 8900-104, Gibco, Waltham, MA, United States), ATCC (PCS-500-012, Lot No. 80124170, ATCC, Manassas, VA, United States) and Lonza (PT-2501, Lot No. 18TL169252, Lonza, Basel, Switzerland), respectively, and used as reference cell lines. The Certificates of Analysis for each cell line, containing the identification of MSC markers, are available on the respective companies’ website. MSCs were cultured in alpha-minimum essential media (α-MEM) containing L-glutamine, ribonucleosides and deoxyribonucleosides (41,061,029, Gibco, Waltham, MA, United States), supplemented with 100 IU penicillin, 100 µg streptomycin and 10% FBS (160,000,044, Thermo Fisher, Waltham, MA, United States**). Since osteoblast-like cells do not proliferate, the number of cells needed for the phenotyping experiments had to be achieved while the cells were still fibroblasts/MSCs/primary cells.

### Osteoblastic differentiation treatment

For the differentiation treatment, the fibroblasts were seeded 10,000–15,000 cells/cm^2^ while the MSCs were seeded 4,000 cells/cm^2^. The differentiation treatment was initiated 24 h after the cells were plated. MSCs confluency was 70–80% at maximum in order not to inhibit their proliferation ability. The fibroblasts were treated for 35 days while the MSCs were treated for 28 days. Differentiation media for fibroblasts consisted of advanced minimum essential media (advanced MEM) (12,492,013, Gibco, Waltham, MA, United States), 1% glutamax, 1% penicillin/streptomycin, 10% FBS*, 0.3 mM L-ascorbic acid (A4544-25 g, Sigma, Saint Louis, MO, United States), 10 mM *ß*-glycerophosphate (G9422-50g, Sigma, Saint Louis, MO, United States) and 100 nM (Day 0–7) or 10 nM (Day 7–35) dexamethasone (D8893-1 MG, Sigma Aldrich, Saint Louis, MO, United States). MSCs were differentiated in media containing α-MEM, 1% glutamax, 1% penicillin/streptomycin, 10% FBS**, 0.05 mM L-ascorbic acid, 10 mM *ß*-glycerophosphate and 100 nM (Day 0–7) or 10 nM (Day 7–28) dexamethasone. The differentiation protocols are illustrated in Supplement 1 (Supplementary Material S1). Non-treated cells were used as controls. Control cells were cultured in their respective growth media (α-MEM, DMEM) until confluence (Day 0).

### Validation of osteoblastic differentiation and mineralization

Osteoblastic differentiation was determined by detecting the preosteoblastic marker ALP and by various mineralization assays at several time points during the osteoblastic treatment. In MSCs and fibroblasts, bone-specific ALP (bALP) activity was measured with immunoenzymometric Ostase® BAP EIA assay (Immunodiagnostic Systems, Holdings Ltd., Boldon, United Kingdom) at 0, 7, 14, 21 days and 0, 7, 14, 21, 28 days, respectively. At each time point, the cells were washed with phosphate-buffered saline (PBS), lysed with Tris-Triton buffer (0.1 M Tris-base, 0.2% Triton X-1000, pH 7) and frozen at −80°C. Prior to measurements, the cell lysates were thawed and centrifuged (12,000 × g, 5 min). bALP was analyzed from aspirated supernatant according to manufacturer’s instructions. ALP was normalized to total protein content (units/mg total protein). At corresponding time points, the cells were fixed with citrate-acetone-formaldehyde solution (26% of citrate, 66% of acetone, 8% of 37%-formaldehyde) for ALP staining and executed according to the kit instructions (86R-1KT, Sigma Aldrich, Saint Louis, MO, United States). The plates were imaged with an EVOS XL core microscope (AMEX 1200, Invitrogen, Waltham, MA, United States) and by using a single lens reflex camera on a light table.

Mineralization was assessed by Alizarin Red S (ARS) and Von kossa (VK) staining detecting deposited minerals. For ARS staining, the MSCs were fixed with citrate-acetone-formaldehyde solution, at 0, 21, and 28 days, while the fibroblasts were fixed at 0, 21, 28, and 35 days. After fixation, cells were incubated with 40 mM ARS solution (2,003,999, Sigma, Saint Louis, MO, United States) at RT for 20 min in the dark. The cells were then washed three times with MQ-H_2_O and one time with tap water. For the VK staining, cells were processed with identical fixative at equivalent time points before executed according to the “Silver plating kit acc. to Von Kossa” kit (100362, Sigma Aldrich, Saint Louis, MO, United States). The plates from ARS and VK staining were imaged as for ALP staining. After imaging, the ARS plates were stored at −20°C, before being analyzed according to the Millipore Osteogenesis assay kit (ECM815, Merck Millipore, Burlington, MA, United States).

### Apoptosis assay

Apoptosis was analyzed in MSCs and Fibroblasts at 0, 14, 21, and 28 days by the kit cell death detection ELISA^PLUS^ (11774425001, Roche Diagnostics, Basel, Switzerland). Each MSC and fibroblast line was plated in six replicates in a 24-well plate before differentiation treatment. At each time point, the cells were washed with PBS, lysed and treated according to the kit instructions. The lysates from six wells were combined into two samples. Each cell line was analyzed in two replicates according to the manufacturer’s instructions.

### RNA isolation for bulk RNA sequencing and qRT-PCR

Gene expression was analyzed at mRNA level with bulk RNA sequencing. RNA sequencing results were validated by qRT-PCR. RNA was isolated from MSCs at 0, 7, 14, 21, and 28 days and from the fibroblasts at 0, 7, 14, 21, 28, and 35 days and extracted using the RNeasy mini kit (74104, Qiagen, Hilden, Germany), according to the manufacturer´s protocol, and DNase treated by utilizing the DNA-free™ DNA Removal Kit (AM 1906, Invitrogen, Waltham, MA, United States). The concentration and the integrity of the RNA was confirmed at the Biomedicum Functional Genomic Unit (FUGU) where RNA quality control analyses (TapeStation 42000 analysis and Qubit analysis) were performed (RNA integrity number, RIN >8.4). The bulk RNA sequencing service was completed by NGI (National Genomic Infrastructure) Sweden.

### cDNA synthesis and qRT-PCR

cDNA was synthesized from 1.4 µg of total RNA using the High-Capacity cDNA Reverse Transcription Kit with RNase inhibitor (4374966, Applied Biosystems, Waltham, MA, United States). Gene expression analyses were performed by qRT-PCR, using TaqMan™ Fast Advanced master mix (4444557, Applied Biosystems, Waltham, MA, United States) and the CFX96 Touch Real-Time PCR Detection System (1845097, BioRad, Berkeley, CA, United States) according to previously described protocol (Bustin, 2000; Adibkia et al., 2021). TaqMan® Gene Expression Assays with FAM dye labeling the probe (Applied Biosystems, Waltham, MA, United States) were used for each gene of interest and the reference genes. Genes of interest; *FGF2* (Hs00266645_m1), *TGFBR2* (Hs00234253_m1), *ALPL* (Hs00758162_m1) and *SERPINF1* (Hs01106937_m1). Reference genes; *TBP* (HS00427620_m1) and *GAPDH* (HS99999905_m1). The gene expressions were normalized to *TBP* and *GAPDH* according to the delta delta Ct method (Livak and Schmittgen, 2001).

### Collection of cells for proteomic analysis

MSCs were collected at 0, 7, 14, 21, and 28 days while fibroblasts were collected at 0, 7, 14, 21, 28, and 35 days. At each time point, the cells were washed with PBS, scraped, rapidly frozen with dry ice, and then stored at −80°C, according to standard protocol (Liu et al., 2018). The cell samples were then processed for MS-analysis and phosphopeptide enrichment.

### Phosphopeptide enrichment

Cells were lysed in 8 M UREA buffer in 50 mM NH4HCO3, sonicated and the cell debris was cleared by centrifugation at 16,000 × g for 20 min. The protein content was measured using a BCA protein assay kit (Pierce, Thermo Scientific, Waltham, MA, United States) and 300 µg of total protein per sample was taken for trypsin digestion. The proteins were reduced with Tris (2-carboxyethyl) phosphine (TCEP; Sigma Aldrich, Saint Louis, MO, United States), alkylated with iodoacetamide and trypsin-digested with Sequencing Grade Modified Trypsin (Promega, Madison, WI, United States) using a 1:100 enzyme:protein ratio at 37°C o/n, and then desalted with C18 spin columns (Nest Group, Cochin, Kerala, India). The sample was divided into two parts: 1/6 (∼50 μg) of the samples were used for total proteome analysis and 5/6 (∼250 μg) were subjected to phosphopeptide enrichment using Ti4+-IMAC. The IMAC material was prepared and used essentially as described (Zhou et al., 2013). Briefly, Ti4+-IMAC beads were loaded onto GELoader tips (Thermo Fisher Scientific, Waltham, MA, United States) and conditioned with loading buffer (80% acetonitrile, ACN, 6% trifluoroacetic acid, TFA). The protein digests were dissolved in a loading buffer and added into the spin tips. The columns were washed first with 50% ACN, 0.1% TFA with 200 mM NaCl and then without salt. The bound phosphopeptides were eluted with 10% ammonia and dried.

### MS-analysis

The LC-MS/MS analysis was performed using a Q Exactive ESI-quadrupole-orbitrap mass spectrometer coupled to an EASY-nLC 1000 nanoflow LC (Thermo Fisher Scientific, Waltham, MA, United States), using Xcalibur version 3.1.66.10 (Thermo Fisher Scientific, Waltham, MA, United States). First, 1/50 of the total proteome samples were injected into a C18-packed pre-column (Acclaim PepMap™100 100 μm × 2 cm, 3 μm, 100 Å; Thermo Fisher Scientific, Waltham, MA, United States) in buffer A (1% ACN/0.1% formic acid, FA). Next, peptides were transferred to a C18-packed analytical column (Acclaim PepMap™100, 75 μm × 15 cm, 2 μm, 100 Å) and separated by a 120-min linear gradient from 5 to 35% of buffer B (98% ACN and 0.1% FA) at a flow rate of 300 nL/min. Finally, the mass spectrometry analysis was performed as DDA in positive-ion mode. MS spectra were acquired from m/z 200 to m/z 2000 with a resolution of 70,000, with full AGC target value of 1,000,000 ions and a maximal injection time of 100 ms in profile mode. The ten most abundant ions with charge states from 2 + to 7+ were selected for subsequent fragmentation (HCD), and MS/MS spectra were acquired with a resolution of 17,500, with an AGC target value of 5,000, a maximal injection time of 100 ms and the lowest mass fixed at m/z 120 in centroid mode. Dynamic exclusion duration was 30 s.

Phosphopeptide samples were resuspended in 15 μl of buffer A of which 8 μL was injected to MS analysis. Peptides were separated by a 120-min linear gradient from 5 to 35% of buffer B (98% ACN and 0.1% FA) at a flow rate of 300 nL/min. MS-parameters were the same as in the total proteome analyses, except MS spectra were acquired from m/z 300 to m/z 2000 with a resolution of 70,000 with Full AGC target value of 3,000,000 ions and a maximal injection time of 120 ms in profile mode.

### RNA-seq, total proteomic and phosphoproteomic analysis

Filtering, normalization, and different analyses, including differential expression analysis, overrepresentation analysis (ORA) and ingenuity pathway analysis (IPA), performed on the RNA-seq, proteomic and phosphoproteomic data by GeneVia Technologies (Tampere, Finland) are described in supplement 1 (Supplementary Material Sl).

### Statistical analysis

ALP and ARS quantification results are presented as mean ± SD (standard deviation) while apoptosis results are presented as mean +SEM (standard error of the mean). Significance was set at *p* < 0.05 and performed with unpaired *t*-test, one tail. Validation of RNA-seq by detecting the relative mRNA expression of *FGF2*, *TGFBR2*, *ALPL* and *SERPINF1* (normalized to *TBP*) was performed with qRT-PCR. The results are presented as mean ± SD. Significance was set at *p* < 0.05 and performed with paired *t*-test, two tailed. The statistical analyses were performed using GraphPad Prism version 8.4.2 for Windows (GraphPad Software, San Diego, CA, United States).

## Results

### Characterization of human MSCs and human dermal fibroblasts

**FIGURE 1:**
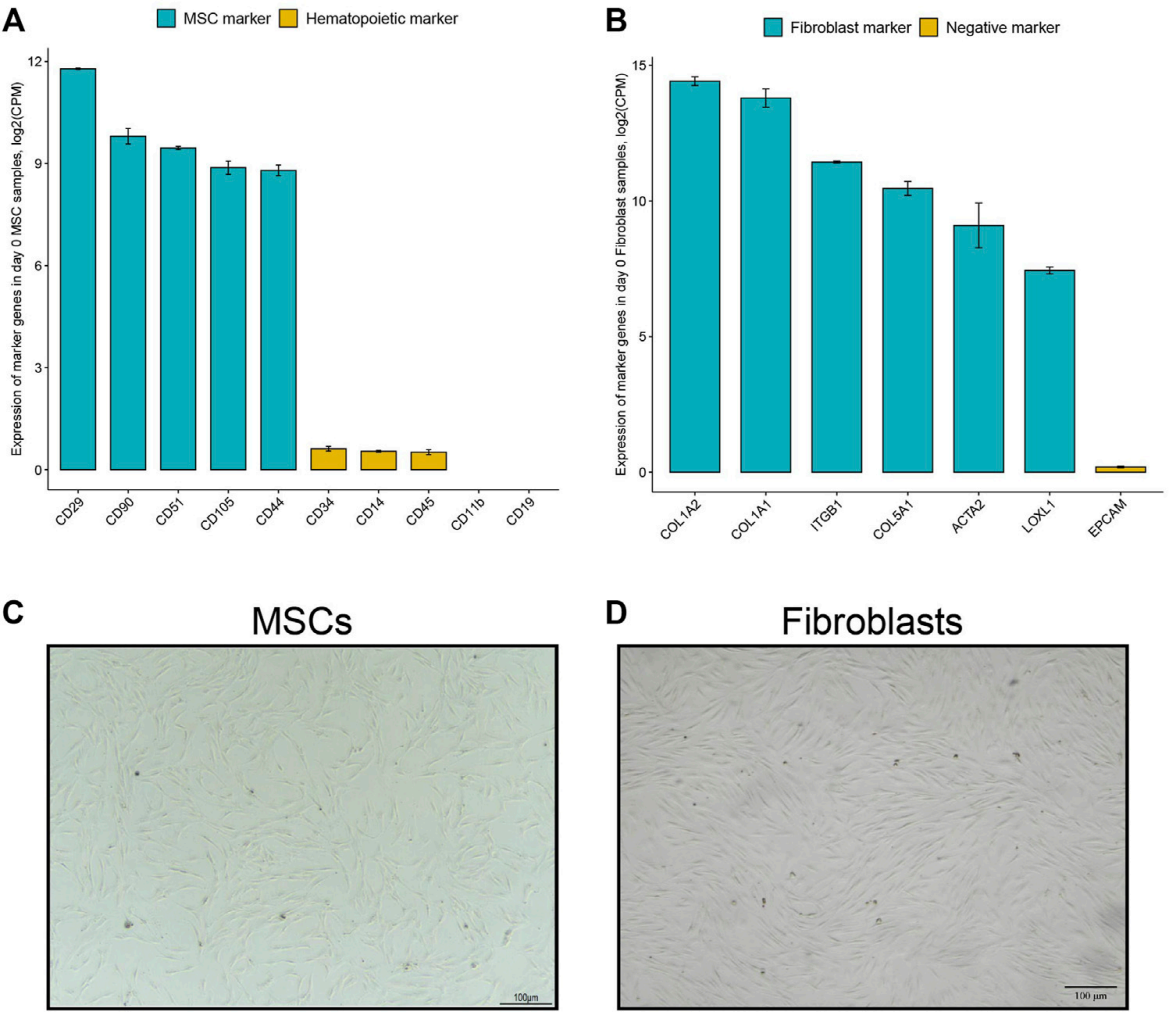
Characterization of MSCs and dermal fibroblasts. **(A)** Marker gene expression in undifferentiated MSCs. Data show expression of known mesenchymal stem cells markers and lack of or low expression of hematopoietic markers. Data are mean +SE (standard error), *n* = 3. **(B)** Marker gene expression in undifferentiated dermal fibroblasts. Data show expression of known fibroblast markers such as collagen genes, ITGB1, ACTA2 and LOXL1, and low expression of fibroblast negative marker EpCAM. Data are mean +SE, n = 4. **(C)** Cell morphology images of undifferentiated MSCs and **(D)** undifferentiated fibroblasts before initiated osteoblastic differentiation treatment. Light microscope, scale bar: 100 µm.

First, we confirmed that the commercial human MSCs displayed the known characteristics described for MSCs (Maleki et al., 2014). Undifferentiated MSCs expressed standard mesenchymal stem cell surface markers and lacked or had only low expression of hematopoietic markers, as shown by RNA-seq data (Figure 1A). Specific molecular markers for dermal fibroblasts are not as clear due to fibroblast heterogeneity. Nevertheless, we detected the gene expression signatures of fibroblast markers *ITGB1*, *ACTA2,* and *LOXL1* (LeBleu and Neilson, 2020; Muhl et al., 2020) by RNA-seq in undifferentiated fibroblasts (Figure 1B). In addition, high expression of three collagen genes (*COL1A2, COL1A1, and COL5A1*) and a low expression of *EpCAM*, an epithelial marker (Olsen et al., 1989; Balzar et al., 1999; Muhl et al., 2020), was detected in undifferentiated fibroblasts (Figure 1B). Furthermore, the morphology of the two cell types was confirmed by light microscopy (Figures 1C,D). Undifferentiated mesenchymal stem cells are characterized morphologically by a small cell body with a few cell processes that are long and thin, while fibroblasts are large, flat, elongated (spindle-shaped) cells possessing processes extending out from the ends of the cell body. These data indicate the cell lineages to be of MSCs and fibroblast origin.

### Osteoblastic differentiation treatment promotes ALP and mineralization but does not affect cell viability

**FIGURE 2:**
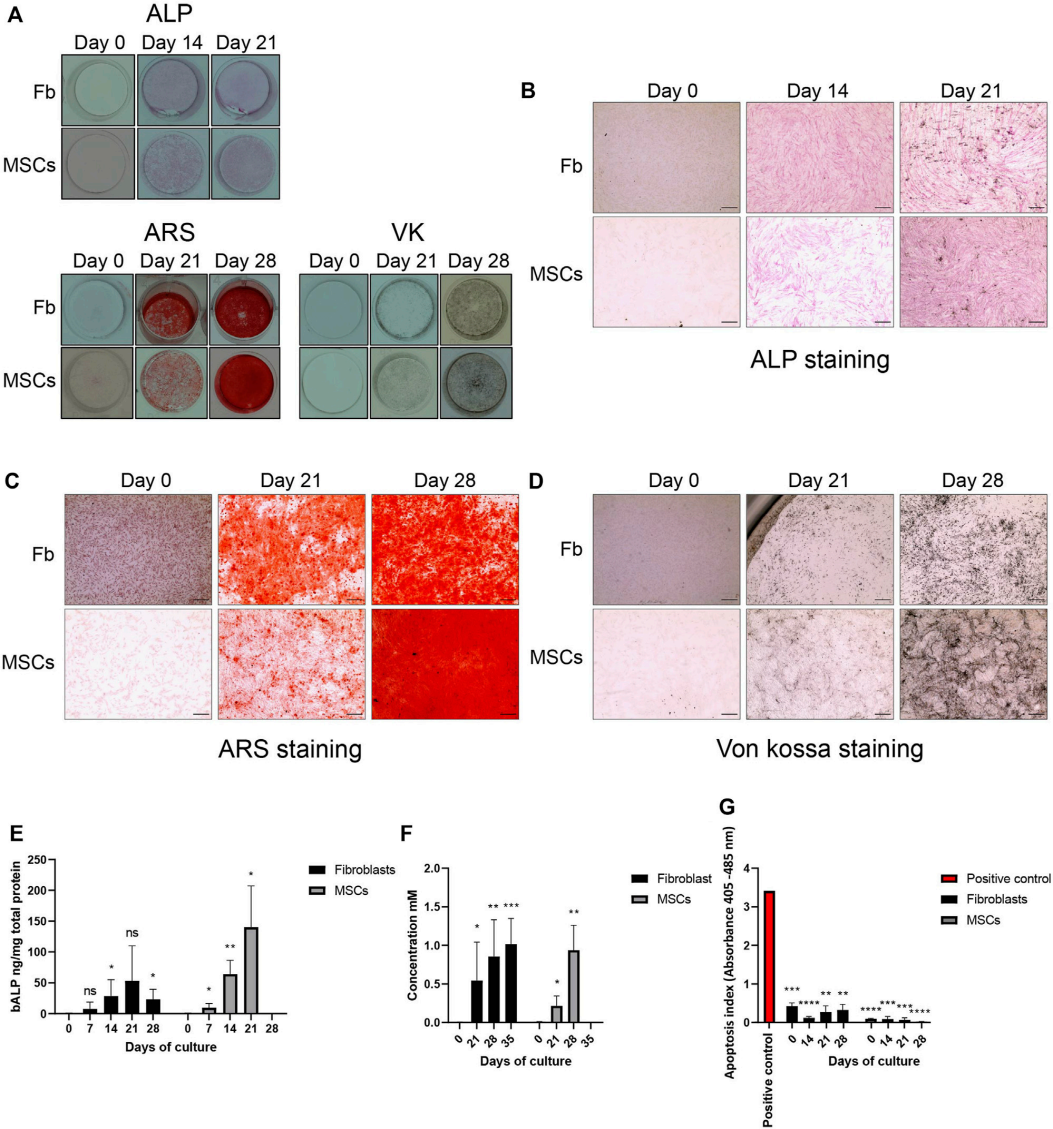
Osteoblastic differentiation. Osteoblastic differentiation of fibroblasts (Fb) and MSCs demonstrated by alkaline phosphatase (ALP, preosteoblastic marker) detection and mineralization staining (Von Kossa, VK), Alizarin Red, ARS). **(A)** An overview of ALP, ARS and VK staining in undifferentiated cells (0 days) and throughout the osteoblastic differentiation treatment (Day 14, 21 and 28). Images were taken by a single lens reflex camera. **(B)** ALP detection at 0 days, 14 days and 21 days. Stained ALP appears in pink. Light microscope, 500 nm scale bar. **(C,D)** Detection of mineral deposits at 0, 21, and 28 days. Calcium deposits stain red with ARS while potassium stains black with VK. Light microscope, 500 nm scale bar. **(E)** Quantified bone ALP (bALP) in fibroblasts and MSCs at 0, 7, 14, 21 and, 28 days and 0, 7, 14 and, 21 days, respectively. Significantly higher bALP can be detected in treated fibroblasts at 14 (*p = 0.0388) and 28 (*p = 0.0146) days compared to undifferentiated fibroblasts. In MSCs, higher bALP can be detected in treated MSCs at 7 (*p = 0.0372), 14 (**p = 0.0040) and 21 (*p = 0.0111) days compared to undifferentiated MSCs. bALP is normalized to total protein content. Mean ± standard deviation (SD). **(F)** Quantification of ARS staining in fibroblasts and MSCs at 0, 21, 28 and 35 days and at 0, 21 and 28 days, respectively. Significantly higher ARS concentration can be detected in fibroblasts treated until 21 (*p = 0.0364), 28 (**p = 0.0057) and 35 (***p = 0.0004) days compared to undifferentiated fibroblasts. Findings were similar in MSCs. Significantly higher ARS concentrations were detected at 21 (*p = 0.0251) and 28 (**p = 0.0038) days treated MSCs compared to undifferentiated MSCs. Mean ± SD. **(G)** Apoptosis. In both fibroblasts and MSCs, low levels of apoptosis were detected in undifferentiated cells and cells treated up to 28 days. There was a significant decrease of apoptosis compared to the positive control (DNA-histone-complex) (**p—****p = 0.0016—< 0.0001). Mean ± SEM. **(E–G)** Unpaired *t*-test, one-tail.

Next, we performed qualitative and quantitative staining analyses to confirm osteoblastic differentiation of fibroblasts and MSCs. Fibroblasts were cultured for 35 days and MSCs for 28 days in osteoblastic differentiation media. After 14 days of treatment, both fibroblasts and MSCs stained positive for ALP and the stain intensified at 21 days (Figure 2B.) The ALP staining was confirmed with a significant increase in bALP in fibroblasts and MSCs, compared to untreated cells, as the differentiation continued (Figure 2E). After 21 and 28 days of treatment, both cell types deposited minerals as shown by ARS and VK staining, indicating mineralization (Figures 2C,D). A quantification analysis of ARS staining confirmed in both fibroblasts and MSCs a significant enrichment of deposited calcium as the osteoblastic differentiation treatment continued (Figure 2F). The treatment did not affect cell viability. Apoptosis rates were significantly lower at every time point in both cell types during the treatment compared to the positive control (Figure 2G). Taken together, our *in vitro* treatment induced osteoblast-like differentiation of fibroblasts and osteoblastic differentiation of MSCs without affecting cell viability.

### Visualization of subgroups in the RNA-seq, proteomic, and phosphoproteomic datasets

**FIGURE 3:**
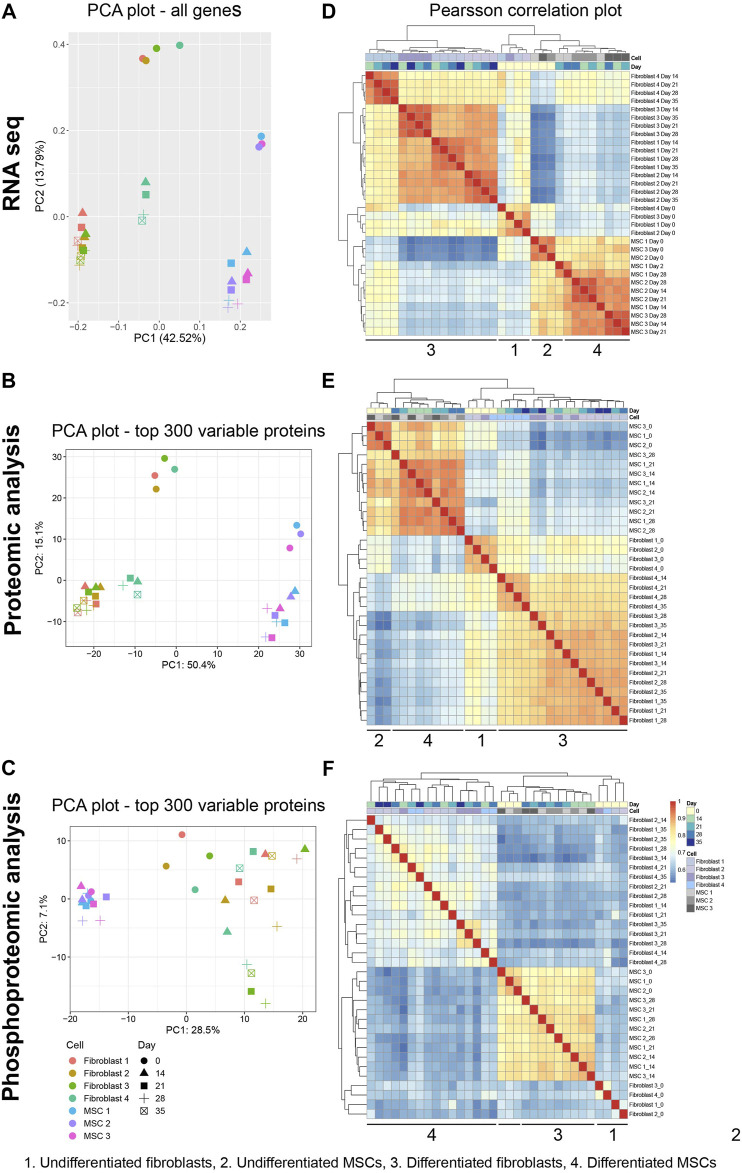
Assessing inter- and intragroup variability of fibroblasts and MSCs. Principal component analysis (PCA) plot visualized all 32 samples in the dataset from all approaches along PC1 and PC2. **(A)** RNA-seq data describe 42.52% (PC1) and 13.79% (PC2) of the variability of all genes. **(B,C)** Data set from protein proteomic analysis describes 50.4% (PC1) and 15.1% (PC2) of variability while from phosphoprotein proteomic the variability was 28.5% (PC1) and 7.1% (PC2) in the top 300 proteins. **(D–F)** Between-sample correlations. Heat maps show the pairwise Pearson’s correlation coefficient. The scale bar of the legends represents the range of the correlation coefficient presented. PCA of data visualize the inter- and intragroup variability between the 32 samples (four fibroblast cell lines at five time points and three MSC lines at four time point). Pearson´s coefficient, describes the directionality and strength of the relationship between two samples.

Differentiation process of fibroblasts and MSCs towards osteoblast-like cells was analyzed in detail by using a multi-omics approach (transcriptomics, proteomics, and phosphoproteomics). In all these three datasets, four groups were distinguished; untreated MSCs and fibroblasts and differentiated MSCs and fibroblasts. In RNA-seq, the group of untreated MSCs was an independent group but closely related to the group of differentiated MSCs, while untreated fibroblasts and differentiated fibroblasts formed two distinct groups (Figures 3A,D). However, one biological replicate of the differentiated fibroblasts deviated from the rest (Fibroblast 4) (Figures 3A,D). Similar group dynamics were also distinguished in proteomic data with a slightly more dispersion in the phosphoproteomic data (Figures 3B,C,E,F). According to Principle Component Analysis (PCA) and Pearson´s coefficient in 32 samples (four fibroblast cell lines at five time points and three MSC lines at four time points) the dataset comprised of four groups; untreated MSCs, untreated fibroblasts, differentiated MSCs and differentiated fibroblasts (Figure 3).

### General expression profiles in fibroblasts and MSCs

**FIGURE 4:**
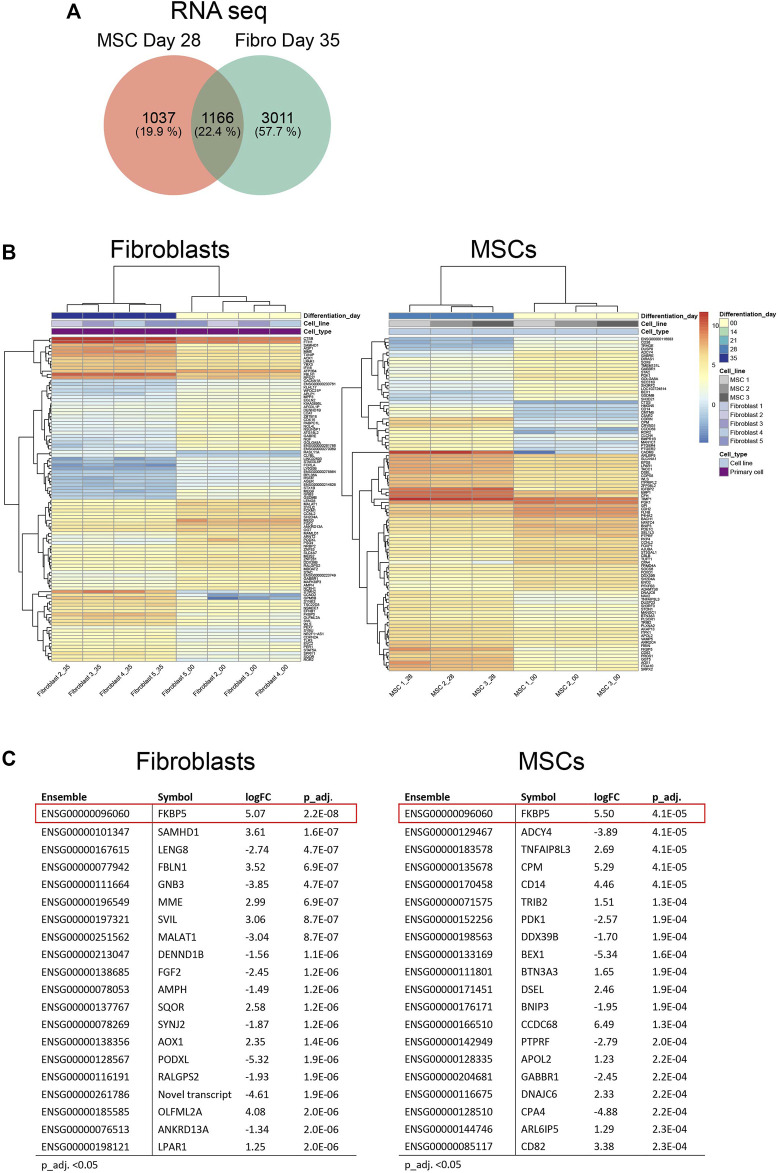
Differential gene expression in fibroblasts and MSCs at endpoint. **(A)** Venn diagram comparing total number of differentially expressed genes in fibroblasts and MSCs, undifferentiated state (Day 0) *versus* differentiated state (Day 35 in fibroblasts/Day 28 in MSCs). In both cell types, 1,166 genes were included, 1,037 genes were exclusively expressed in MSCs while 3,011 genes were exclusively expressed in fibroblasts. **(B)** Top 100 and **(C)** Top 20 differentially expressed genes in MSCs and fibroblasts comparing undifferentiated state to last time point. FKBP5, highlighted in red, was differentially expressed in both cell types.

**FIGURE 5:**
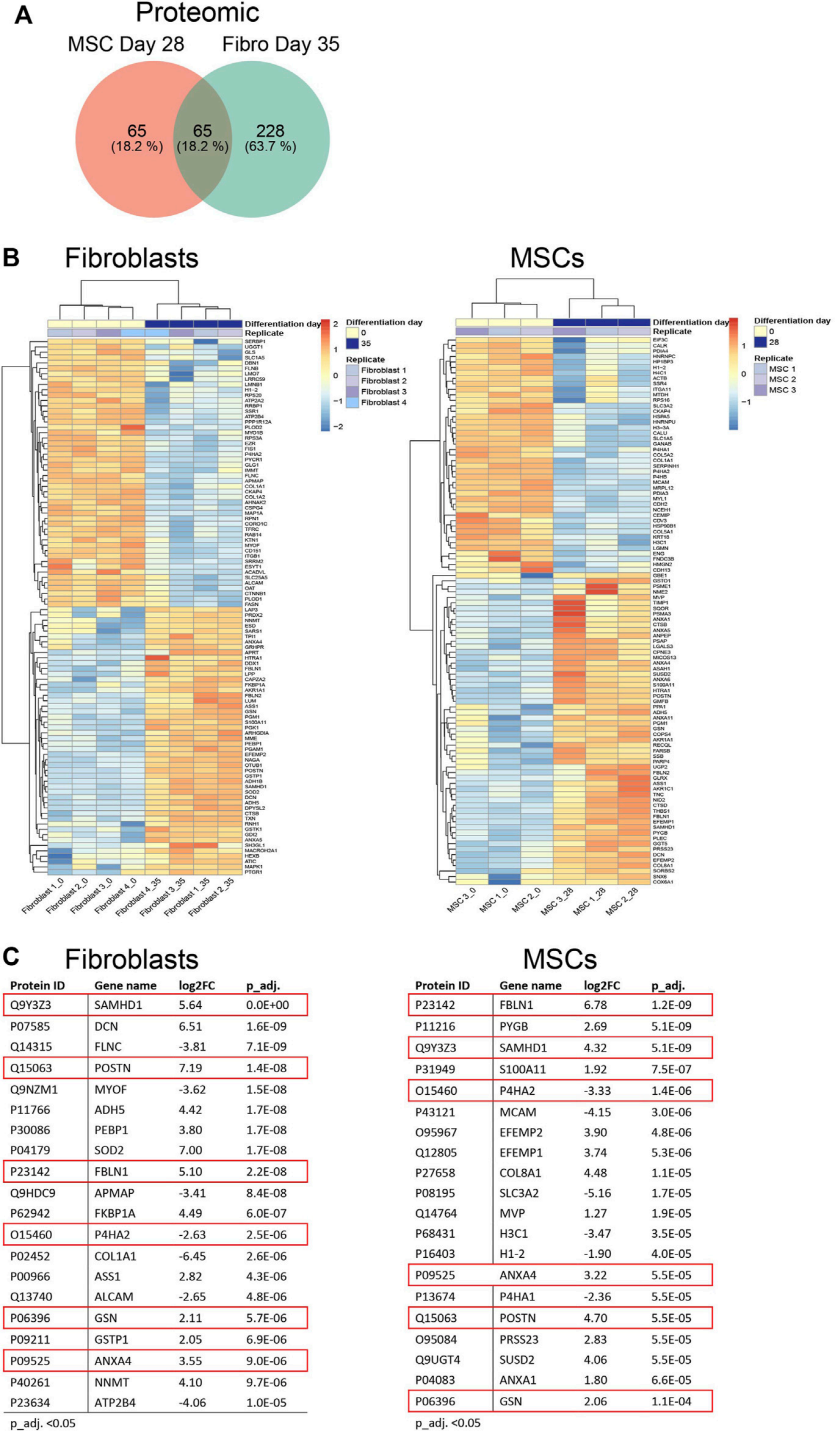
Differential protein expression in fibroblasts and MSCs at endpoint. **(A)** Venn diagram comparing total number of differentially expressed proteins in fibroblasts and MSCs, undifferentiated state (Day 0) *versus* differentiated state (Day 35 in fibroblasts/Day 28 in MSCs). In both cell types, 65 proteins were included, 65 proteins were exclusively expressed in MSCs while 228 proteins were exclusively expressed in fibroblasts. **(B)** Top 100 and **(C)** Top 20 differentially expressed proteins in MSCs and fibroblasts comparing undifferentiated state to last time point. FBLN1, SAMHD1, P4HA2, POSTN, ANXA4, GSN, highlighted in red, were differentially expressed in both cell types.

We continued to investigate the expression profiles in MSCs and fibroblasts during the osteoblastic differentiation treatment. In total, RNA-seq analysis identified 18,469 transcripts covering 15,799 genes used for differential expression analysis. Proteome-profiling identified 3,091 peptides of which 1,235 passed filtering and were used for differential analysis. In addition, we identified a total of 4,827 phosphosites, representing 1,798 phosphoproteins; 1,806 phosphosites in 903 unique proteins were retained for differential analysis after filtering (explained in Supplementary Material S1). Differential expression analysis (adjusted *p*-value (p_adj.) <0.05) in fibroblasts and MSCs, comparing the undifferentiated state (time point 0) to the last time point (28 days in MSCs and 35 days in fibroblasts), showed 1,166 differentially expressed genes in both cell types, 1,037 genes exclusively expressed in MSCs and 3,011 genes exclusively expressed in fibroblasts (Figure 4A; Supplementary Material S3A). Most of the 1,166 genes, which were differentially expressed in both cell types, showed a similar up- and down-regulation profile when comparing the gene expression in the two cell types and only 27 genes differed (Supplementary Material S3B). Figure 4B summarizes the top 100 most differentially expressed genes based on the cutoff p_adj. <0.05 in the end-point analysis of fibroblast and MSCs (Figure 4B). Expression profiles were highly similar between the respective biological replicates in both cell types. However, the most differentially expressed genes differed notably between cell types. Out of the top 20 most differentially expressed genes, only *FKBP5* was identified in both fibroblasts and MSCs (Figure 4C). *FKBP5* encodes a member of the immunophilin protein family, which plays a role in immunoregulation and in essential cellular processes involving protein folding and trafficking (Harikishore and Yoon, 2015). *FKBP5* is also a strong glucocorticoid response gene and its activation is most likely due to added dexamethasone (Bancos et al., 2021). In contrast to the RNA-seq data, the differentially expressed proteins showed higher similarities between the cell types. Differential expression analysis (p_adj. <0.05) in fibroblasts and MSCs, comparing time point 0 to 28 days in MSCs and to 35 days in fibroblasts, showed 65 differentially expressed proteins in both cell types, 65 proteins only differently expressed in MSCs and 228 proteins only differently expressed in fibroblasts (Figure 5A; Supplementary Material S3C). When comparing the protein expression of the 65 differentially expressed proteins in both cell types, similar up- and down-regulation profiles were observed in both cell types, with only 4 proteins that were regulated differently (Supplementary Material S3D). In addition, the top 100 most differentially expressed proteins in MSCs and fibroblasts from the undifferentiated state to the last time point, showed high similarity in expression profiles between the respective biological replicates (Figure 5B). In the top 20 differentially expressed proteins, a total of six proteins were differentially expressed in both cell types, including SAMHD1, POSTN, FBLN1, P4HA2, GSN, and ANXA4 (Figure 5C). This indicates that similar osteoblastic treatments induce similarities in the two *in vitro* cell model systems but also provoke differences, which is expected when the starting material differs.

### Similar gene and protein expressions patterns in fibroblasts and MSCs

**FIGURE 6:**
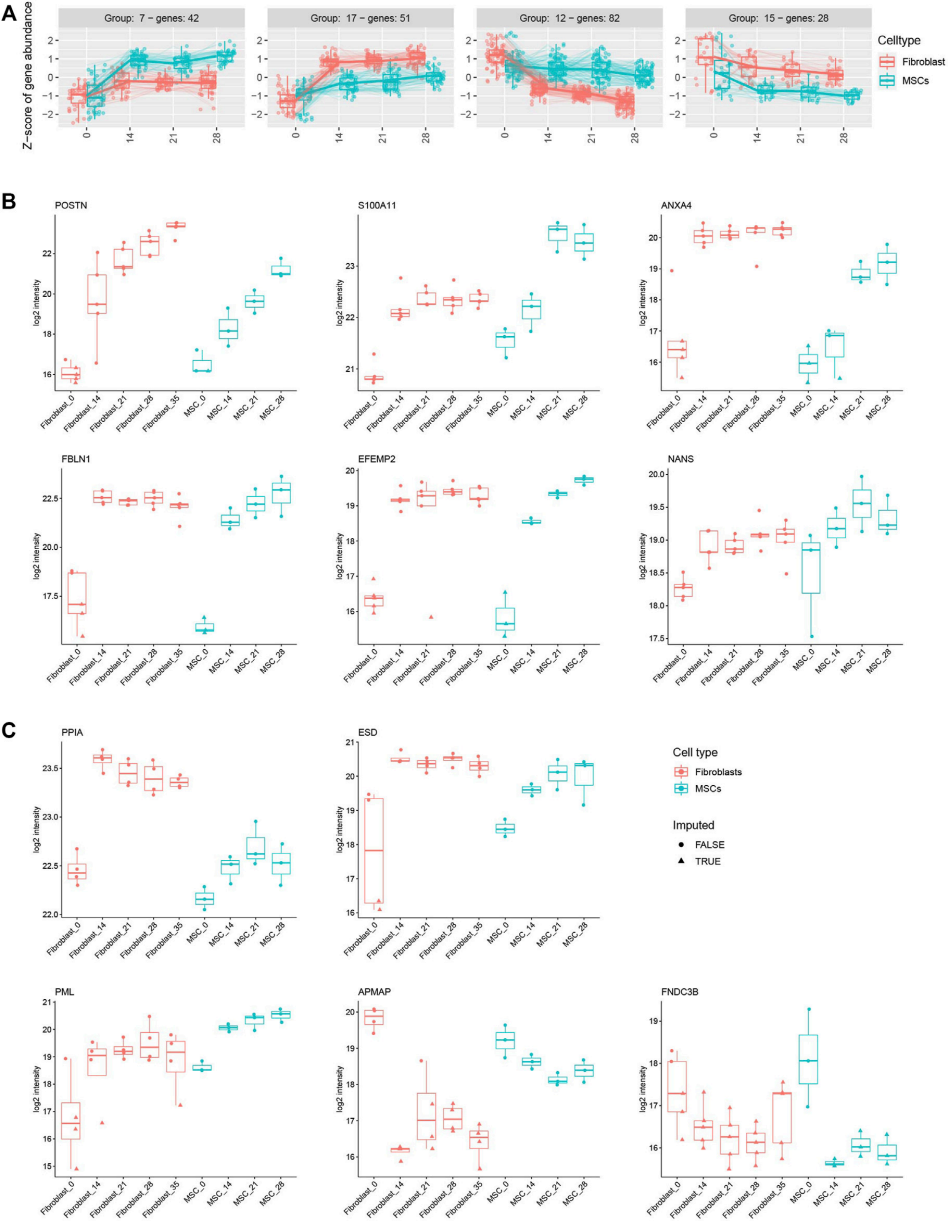
Osteoblastic differentiation treatment induces similar gene and protein profiles in fibroblasts and MSCs. **(A)** Four clusters of up and down-regulated genes in both cell types during the osteoblastic differentiation. In total, 42 genes in group 7 and 51 genes in group 17 are up-regulated while 82 genes in group 12 and 28 genes in group 15 are down-regulated. Clusters include all significantly differentially expressed genes with a p_adj. <0.05 in any of the time points from the course analysis. **(B)** Protein expression of POSTN, S100A11, ANXA4, FBLN1, EFEMP2, and NANS in fibroblasts and MSCs during the osteoblastic treatment. The proteins are significantly differently expressed (p_adj. <0.05) in both cell types. **(C)** Protein expression of PPIA, ESD, PML, APMAP (significantly differentially expressed in fibroblasts, p_adj. <0.05) and FNDC3B (significantly differentially expressed in MSCs, p_adj. <0.05) in both cell types during the treatment. Box plots showing the VSN-normalized protein intensities of each of the differently expressed proteins across all fibroblast and MSC samples were generated, indicating imputed and non-imputed values with symbols.

Next, we utilized time-course differential expression analysis to display groups of similarly expressed genes in both cell types during the osteoblastic differentiation treatment. For clustering, all genes that were significantly differentially expressed with p_adj. <0.05 at any time points (from Day 0 to 7, 14, 21, 28, or 35 Days) from the time-course analysis were taken as an input. A total of 1,024 genes were distributed across 20 clusters of various sizes (Supplementary Material S2, Figure S1A; Supplementary Figure S3E), visualizing both up- and down-regulated genes and creating a larger scale view of the differentiation process. For example, groups 7 and 17 shared up-regulated genes in both cell types during the osteoblastic differentiation while groups 12 and 15 showed clusters of genes with decreased expression (Figure 6A). From the up-regulated gene cluster 7, we identified *OMD*, *JAK2*, *MMP3* and *CSFI* while from up-regulated gene cluster 17 we identified *CLIP* and *SOD2*. From the down-regulated gene cluster 12, we identified *SOX9*, *APCDD1L*, *NCEH1* and *LRP8* while from down-regulated gene cluster 15 we identified *PDK1*. The selected genes identified in the data set and their function in osteogenic differentiation are listed in Table 1. The complete gene lists of all four clusters are included in the supplements (Supplementary Material S2, Figure S1B).

**TABLE 1:** Genes, proteins, and phosphoproteins associated with osteogenesis.

Gene	Function in osteogenic differentiation	Regulation
CLIP	Novel regulator of matrix mineralization (Staines et al., 2014)	↑
SOD2	Eliminator of excess mitochondrial superoxide and protein oxidation that is needed for osteoblastic differentiation and bone formation (Gao et al., 2018)	↑
OMD	Involved in osteogenic differentiation (Lin et al., 2021)	↑
JAK2	Involved in mineralization processes (Yu et al., 2018)	↑
CSFI	Osteogenesis marker. Osteoblasts express at least four transcripts encoding either a secreted or a membrane-bound form of CSF-1 (Cecchini et al., 1997)	↑
SOX9	Inhibitor of osteoblast development (Loebel et al., 2015)	↓
APCDD1L	Function as a negative regulator of the critical bone related WNT signaling pathway (Shimomura et al., 2010)	↓
NCEH1 & LRP8	Associated with cholesterol metabolism. Cholesterol may inhibit vital osteoblastic genes in osteoblast cells, which in turn inhibits osteoblastic differentiation (Dolley et al., 2011; Zhang et al., 2017; Yin et al., 2019)	↓
MMP3	Inhibitor of osteoblastic differentiation (Zhao et al., 2018)	↓
PDK1	Associated with proliferation. An increased differentiation cause a decrease in proliferation (Nakamura et al., 2008; Ruijtenberg and van den Heuvel, 2016)	↓
ALP	Essential inducer of osteoblastic differentiation (Bolger, 1975)	↑
RUNX2	Vital inducer of osteoblast formation (Loebel et al., 2015)	↑
COL15A1	EMC organizer in the early phase of osteogenesis (Lisignoli et al., 2017)	↑
**Protein**	**Function in osteogenic differentiation**	**Regulation**
POSTN	Cell adhesion molecule for preosteoblasts and is involved in the recruitment, attachment and spreading of osteoblast (Kruzynska-Frejtag et al., 2001)	↑
S100A11	Novel marker of osteogenic differentiation (Granéli et al., 2014)	↑
ANXA4	Novel marker of osteogenic differentiation (Granéli et al., 2014)	↑
FBLN1 (also gene)	Required for bone formation and Bmp-2-mediated stimulation of Osterix, inducing osteoblastic differentiation (Cooley et al., 2014; Hang Pham et al., 2017)	↑
EFEMP2	Expressed in mouse osteoblasts and controls collagen fibril assembly, an important process in bone development (Papke et al., 2015)	↑
NANS	Required for the synthesis of sialic acid and the activity of NANS is needed for incorporating sialic acid precursors into sialylated glycoproteins, ensuring a proper skeletal development (van Karnebeek et al., 2016)	↑
ANXA1,5,6,11	Role in in proliferation and osteogenic differentiation (Granéli et al., 2014; Pan et al., 2015)	↑
PPIA	BMP-2 induced phosphorylation of Smad1/5/8 needed in the regulation of osteoblastic activity (Guo et al., 2016)	↑
ESD	Involved in the recycling of sialic acid. Sialic acid is needed for the expression of important bone mineralization factors such as of bone sialoprotein (BSP), osteoprotegerin (OPG), and vitamin D receptor (VDR) (Xu et al., 2013)	↑
PML (also phos. protein)	Regulates hMSCs as an inhibitor of cell proliferation but as a promoter of osteogenic differentiation (Sun et al., 2013)	↑
APMAP	Play a role in adipocyte differentiation (Mosser et al., 2015)	↓
FNDC3B	Play a role in adipocyte differentiation and a negative regulator of osteoblastic differentiation (Kishimoto et al., 2010)	↓
**Phos. protein**	**Function in osteogenic differentiation**	**Regulation**
PTRF	Abundant protein in osteoblasts (Kopanos et al., 2019)	↑
CHAMP2B	Important in the intracellular trafficking of osteoblasts (Aasebø et al., 2021)	↑

Based on the differential expression analysis (p_adj. <0.05), presented in Figures 5A, six proteins, POSTN, S100A11, ANXA4, FBLN1, EFEMP2 and NANS that all associate with bone developmental processes, showed a significant increase in expression in both cell types during the osteoblastic differentiation (Figure 6B; Table 1). In addition, other Annexins such as ANXA1, ANXA5, ANXA6, ANXA11, with a role in osteogenic differentiation, also showed significantly elevated expressions (Supplementary Material S2, Figure S2; Table 1). Moreover, five important osteogenic proteins were differentially expressed during the osteogenic differentiation in both cell types: PPIA, ESD, and PML were increased while APMAP was decreased (Figure 6C; Table 1). However, the changes in the expression were significant only in fibroblasts (p_adj. <0.05). Similarly, FNDC3B expression was decreased after the differentiation treatment in both cell types but reached statistical significance only in MSCs (p_adj. <0.05) (Figure 6C; Table 1). A full list of all differentially expressed proteins during the differentiation process in fibroblasts and MSCs is included in the supplement (p_adj. <0.05) (Supplementary Material S3F).

**FIGURE 7:**
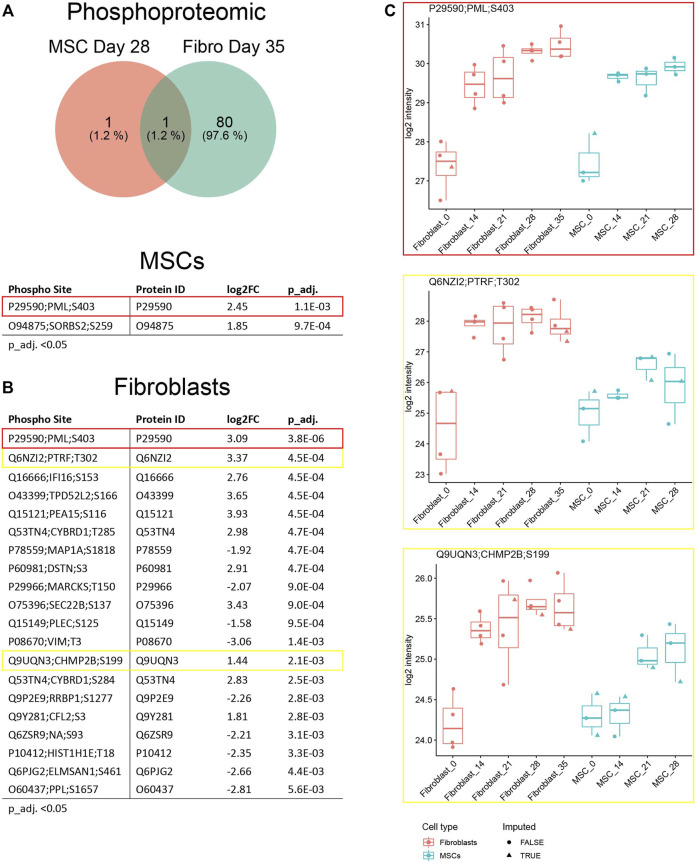
Phosphoproteomic profiles in fibroblasts and MSCs induced by osteoblastic differentiation. **(A)** Total number of differentially expressed phosphorylated protein sites in fibroblasts and MSCs, undifferentiated state *versus* differentiated state. One phosphorylated protein included in both cell types, one exclusively in MSCs and 80 exclusively in fibroblasts. **(B)** Top 20 most differentially expressed phosphorylated proteins in MSCs and fibroblasts comparing undifferentiated state to last time point, p_adj. <0.05 (Benjamini–Hochberg FDR adjustment) **(C)** Hyperphosphorylation of PML (marked in red), PTRF (yellow) and CHMP2B (yellow) during the osteoblastic differentiation in both cell types. Box plots of intensity values of the differently expressed phosphoproteins (p_adj. <0.05) from each comparison were plotted, showing the imputation status of each intensity value.

Analysis of changes in phosphorylated protein expressions during osteoblastic differentiation, based on cutoff p_adj. <0.05, when comparing baseline to 28 days in MSCs and to 35 days in fibroblasts, showed only one differentially expressed phosphorylated protein shared by both cell types, only one phosphorylated protein exclusive to MSCs and 80 phosphorylated proteins exclusive to fibroblasts (Figure 7A; Supplementary Material S3G). Top 20 of these proteins are illustrated in Figure 7B. One of the highlighted proteins, PML, was hyperphosphorylated on position S403 in both cell types and the phosphorylation increased steadily as the treatment continued (Figure 7C; Table 1). As mentioned, elevated expression of PML was also found on protein level (Figure 6C). Two other proteins, PTRF and CHMP2B, showed an increased phosphorylation in both cell types during the osteoblastic differentiation, but the increase was statistically significant only in fibroblasts (Figures 7B,C; Table 1).

Together, these results indicate that the osteoblastic differentiation treatment modulates genes, proteins, and protein phosphosites that can be linked to osteogenesis or associated processes in both fibroblasts and MSCs (Figures 6, 7).

### Down-regulation of cell-type specific markers and up-regulation of osteoblast markers during differentiation

**FIGURE 8:**
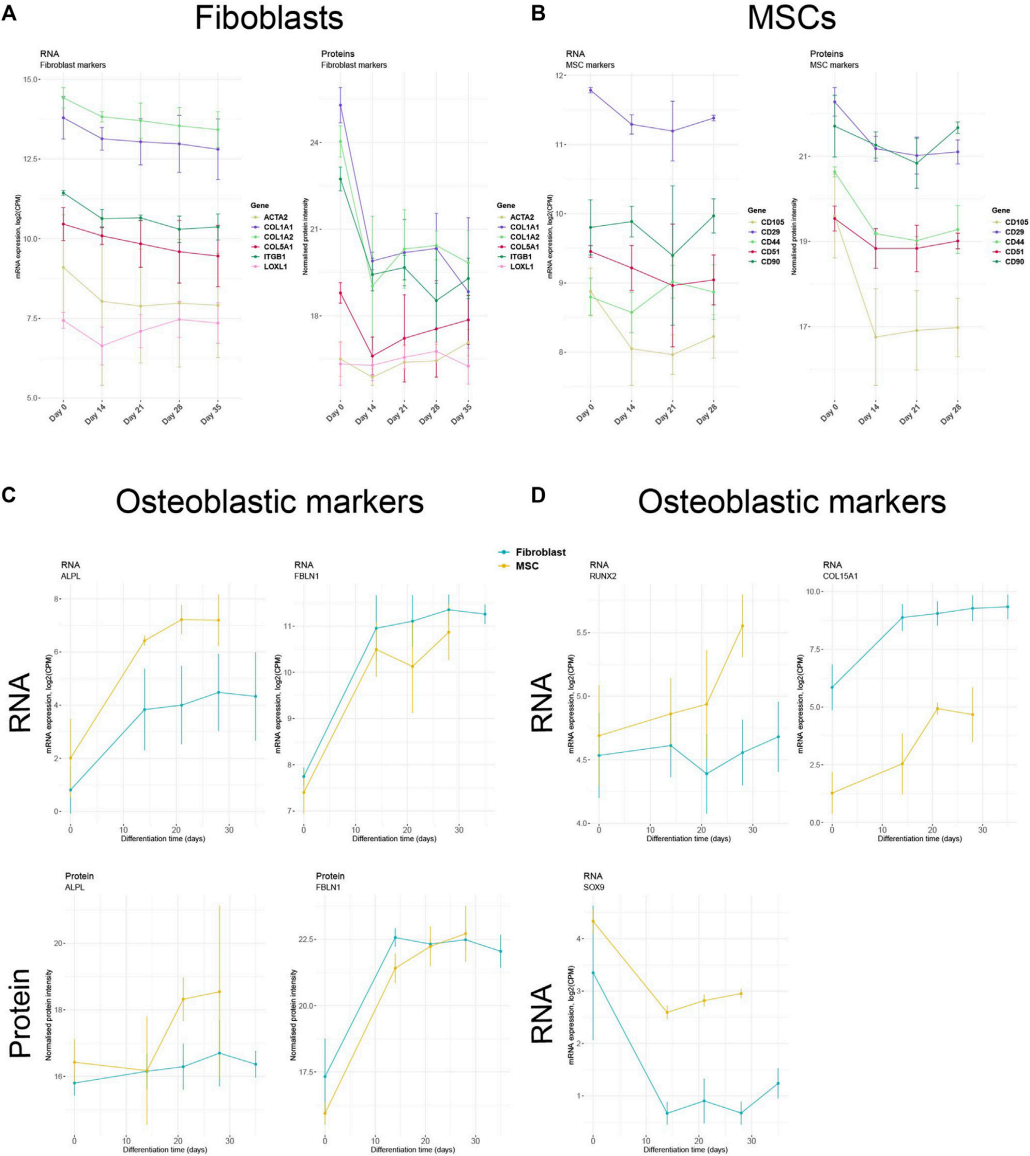
Decreased expression of cell type-specific markers and increased expression of osteoblast markers **(A)** Expression profiles of selected fibroblast markers in fibroblasts and **(B)** selected MSC markers in MSCs during osteoblastic differentiation treatment. **(C)** RNA and protein or **(D)** only RNA expression of essential osteoblastic markers in MSCs and fibroblasts. For mRNA expression plots, log2-transformed counts per million (CPM) values were used. For protein expression plots, normalized and imputed proteomics data were used.

To further validate the differentiation protocol, we analyzed the expression of standard fibroblast and MSC markers prior to the treatment and during the osteoblastic differentiation. Fibroblast markers such as COL1A1, COL1A2, COL5A1, ACTA2, ITGB1, and LOXL1 peaked before the initiation of the osteoblastic treatment (Day 0) and plateaued during the differentiation on both RNA and protein levels (Figure 8A). A similar trend was detected in MSCs. The known MSC markers CD105, CD29, CD44, CD51, and CD90 were persistently expressed in undifferentiated MSCs (Day 0) with a decrease in RNA and protein expression as the differentiation towards osteoblastic-like phenotype advanced (Figure 8B). The observed down-regulation indicates that the cells lose their cell-typical identity and differentiate towards another cell type. Therefore, we analyzed the RNA and protein levels of several genes and proteins fundamental for osteogenesis. On both RNA and protein level, the expression of ALPL (inducer of osteoblastic differentiation) and FBLN1 (positive modulator of bone formation) (Bolger, 1975; Cooley et al., 2014) increased simultaneously as the expression of fibroblast- and MCS- specific markers plateaued (Figure 8C). Increased ALPL expression was however indistinct due to high error bars. In addition, RNA-seq data showed in both cell types a vague elevated expression of *RUNX2*, a vital inducer of osteoblast formation (Loebel et al., 2015), as the treatment continued (Figure 8D). The gene profile of *SOX9*, a suppressor of osteogenesis (Loebel et al., 2015), was down-regulated while levels of *COL15A1*, an EMC organizer in the early phase of osteogenesis (Lisignoli et al., 2017), was up-regulated during the treatment in both cell type (Figure 8D). The gene profile of the cells thus changed towards a more osteoblast-like profile during the differentiation treatment.

The RNA-seq data were validated by confirming by qRT-PCR the mRNA expression of four target genes. In Supplementary Material S2, Figure S3, we illustrate down-regulation of *FGF2*, similarly detected by both qRT-PCR and RNA-seq, in fibroblasts and MSCs as the osteoblastic differentiation treatment continued (Supplementary Material S2, Figure S3). Also, an up-regulation of *ALPL*, *TGFBR2* and *SERPINF1* was detected by both qRT-PCR and RNA-seq in both cell types during the osteoblastic differentiation treatment (Supplementary Material S2, Figure S3). These results validate the presented RNA-seq findings.

### Gene profile of known osteogenic genes during osteoblastic differentiation treatment

**FIGURE 9:**
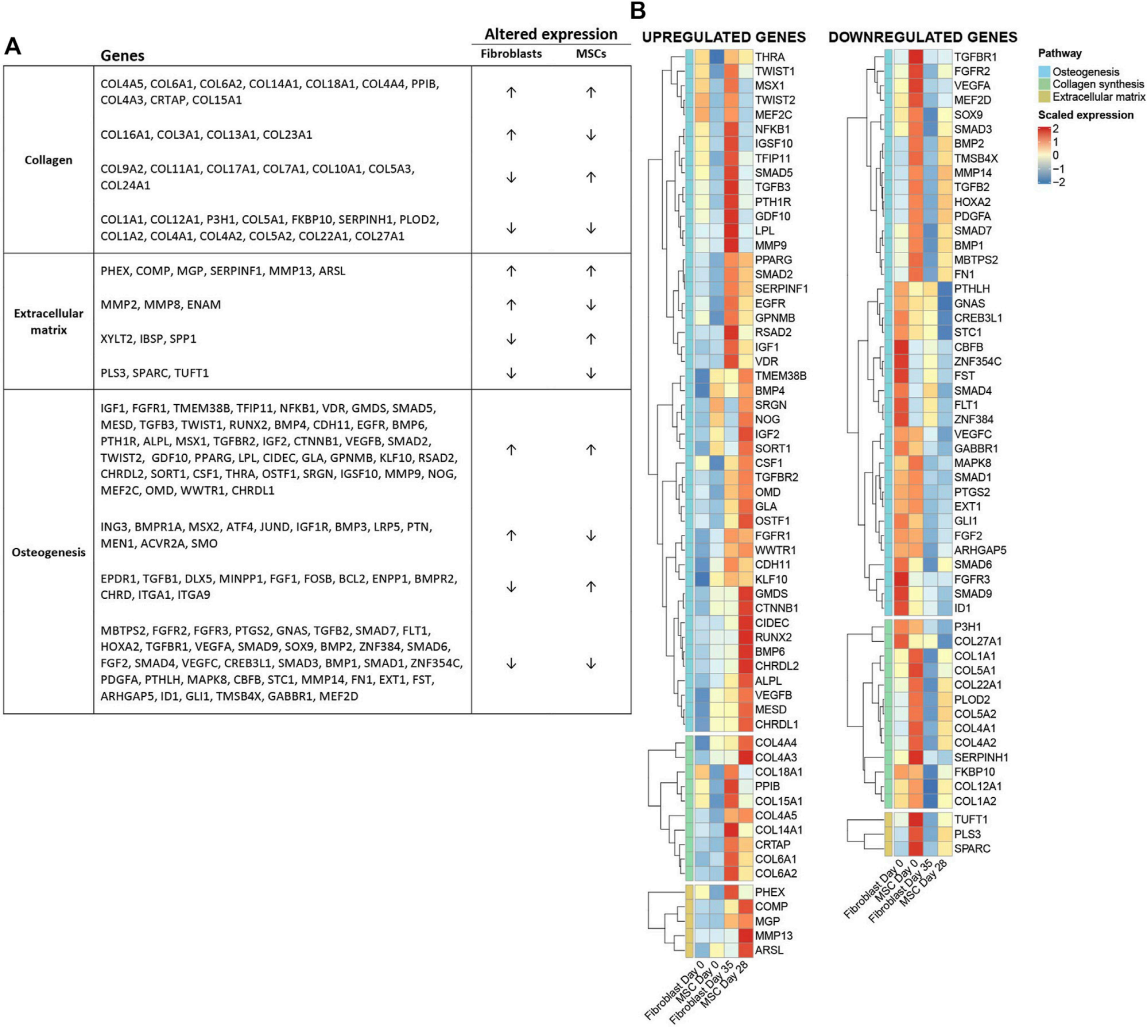
Gene profile of known osteogenesis-linked genes during osteoblastic differentiation treatment. **(A)** List of 158 genes divided into three essential classes of bone development: collagen genes, extracellular matrix genes and osteogenesis genes. Overall, 62 genes were up-regulated in both cell types (↑↑), 41 genes were altered differently between the cells types (↑↓ or ↓↑) and 55 genes were down-regulated (↓↓) in both cell types from the first to the last time point during the osteoblastic differentiation treatment. **(B)** The 62 up-regulated genes and the 55 down-regulated genes are illustrated in two heat maps. The heat maps are clustered according to the three above-mentioned classes. RNA dataset filtering and normalization performed with DESeq2 R package; differential expression analysis (DEA) and statistics performed in R using the limma package. Log2FC per gene was calculated based on the group means. Statistical significance of genes was determined by adjusted *p*-value cutoff p_adj. <0.05 (using Benjamini–Hochberg FDR adjustment).

To ensure that our osteoblastic treatment differentiates fibroblasts and MSCs into a more osteoblast-like profile, we analyzed RNA expression of numerous known genes linked to osteogenesis or processes of bone development. The genes were selected based on a literature search. Figure 9A illustrates a list of 158 known osteogenesis-linked genes and their differential expression in MSCs and fibroblast during the osteoblastic differentiation treatment from first to last time point (Figure 9A). In both cell types, 62 genes were up-regulated (↑↑), 41 genes were altered differentially between the cell types (↑↓ or ↓↑) and 55 genes were down-regulated in both MSCs and fibroblasts (↓↓). A handful of known genes linked to osteogenesis did not pass through the filtering of RNA-seq data (Supplementary Material S2, Figure S4). Figure 9B illustrates the 62 up-regulated and 55 down-regulated genes in two heat maps (Figure 9B). The expression of many essential osteogenesis-linked genes were altered according to an osteoblastic phenotype in both cell types during the treatment, suggesting that the osteoblastic differentiation treatment is pushing the cells toward an osteoblast-like cell type.

### Activation of the osteoarthritic pathway in osteoblast-like cells

**FIGURE 10:**
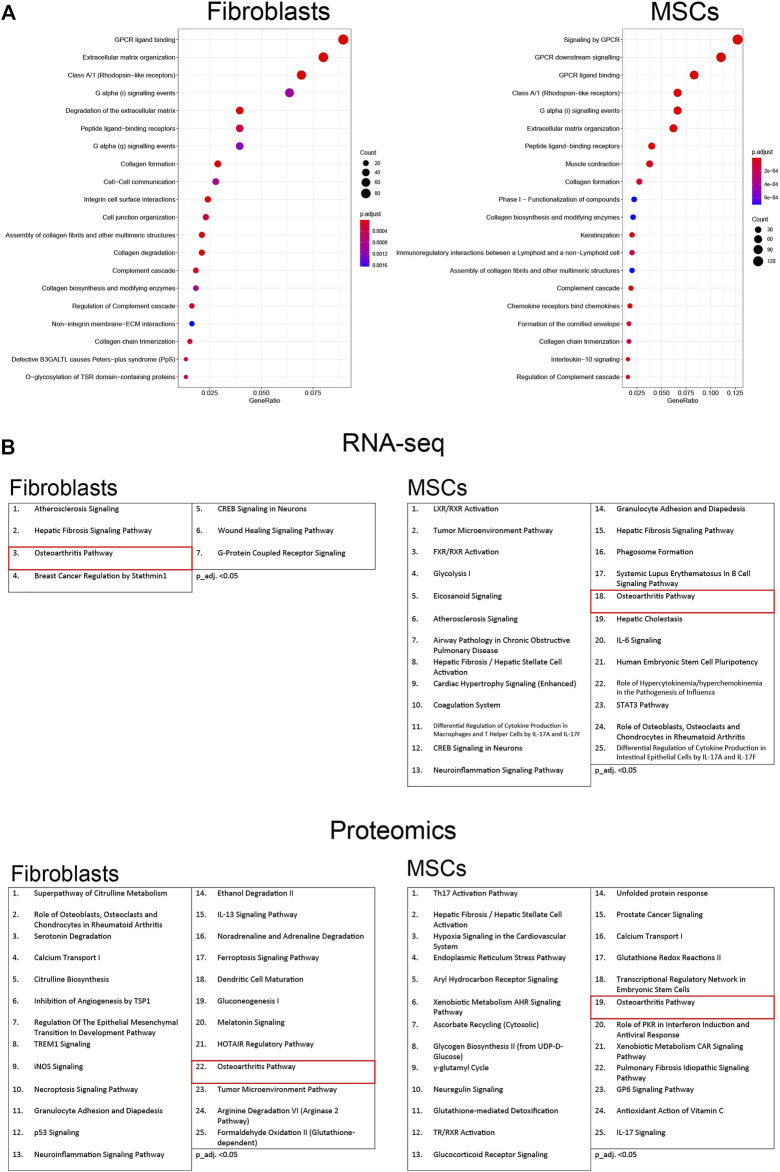
Pathway analysis of osteoblastic differentiated fibroblasts and MSCs. **(A)** The top 20 most significant gene sets, based on the most differentially expressed genes in osteoblastic differentiated fibroblasts and MSCs. Overrepresentation analysis (ORA) was conducted using R package clusterProfiler. Differentially expressed genes for the enrichment analysis were chosen using adjusted *p*-value threshold of 0.05 and requiring at least 1.5-fold up- or down-regulation in expression. The *p*-values of enrichment analysis were adjusted for multiple testing using Benjamini–Hochberg procedure. Enriched terms were further visualized using clusterProfiler functions. **(B)** The top 25 enriched categories of canonical pathways in RNA-seq and in proteomic data (p_adj. <0.05). Pathways are activated in fibroblasts and MSCs from the first to the last time point during the osteoblastic differentiation treatment. The osteoarthritic pathway is marker in red. Used cutoff for IPA analysis in RNA-seq is log2FC > abs (2) and p_adj. <0.01 and in proteomic is log2FC > abs (2) and adj. p.Val <0.05. Adjusted *p*-value 0.05 was used as a threshold for filtering for statistically significant results.

Finally, to acquire a greater perspective on how the osteoblastic differentiation treatment affected the two cell types, the RNA-seq data were analyzed with both Overrepresentation Analysis (ORA) and Ingenuity Pathways Analysis (IPA), and the proteomic data with only IPA. ORA determines whether any terms are annotated to a list of specific genes, in this case a list of differentially expressed genes, at a frequency greater than what would be expected by chance, and a *p*-value was calculated using hypergeometric distribution. The total set of genes from the dataset was used as a list for background genes. ORA analysis was performed on differentially expressed genes of fibroblast at 35 days and MSCs at 28 days. ORA dot plots, visualize the 20 most significant gene sets in differentiated fibroblasts and MSCs (Figure 10A). Gene sets involved in G protein-coupled receptor (GPCR) signaling and ligand binding were altered in both cell types; GPCRs have an essential role in bone formation (Luo et al., 2019). In addition, expression of genes included in extracellular matrix organization and in collagen processes, both fundamental processes in osteogenesis (Lin et al., 2020), were predicted to be regulated. Similarly, expression of complement cascade-associated genes was modulated in both cell types. The complement system is part of innate immunity and thought to be critical for bone growth (Mödinger et al., 2018). The expression details are illustrated in the supplement (Supplementary Material S2, Figure S5).

We also performed over-representation analysis using the IPA framework and their manually curated pathway database (QIAGEN Knowledge Base) to interpret the complex effects of the significantly regulated genes through network analysis, and to elucidate the molecular pathways affected by the osteoblastic differentiation treatment in each cell type from undifferentiated state (Day 0) to the treatment endpoint (Day28 in MSCs/Day35 in Fibroblasts). IPA takes significantly regulated genes as input and investigates enrichment in the manually curated pathway database. The top 25 enriched categories of canonical pathways with p_adj. <0.05 in RNA seq-data and proteomic data are shown in Figure 10B. The “osteoarthritis pathway” was activated in both cell types during the treatment, in line with osteoblastic differentiation (Maruotti et al., 2017). Other pathways activated during the osteoblastic differentiation are the “Role of Osteoblasts, osteoclasts and Chondrocytes in Rheumatoid Arthritis”, “wound healing signaling pathway” and “atherosclerosis signaling pathway”.

**FIGURE 11:**
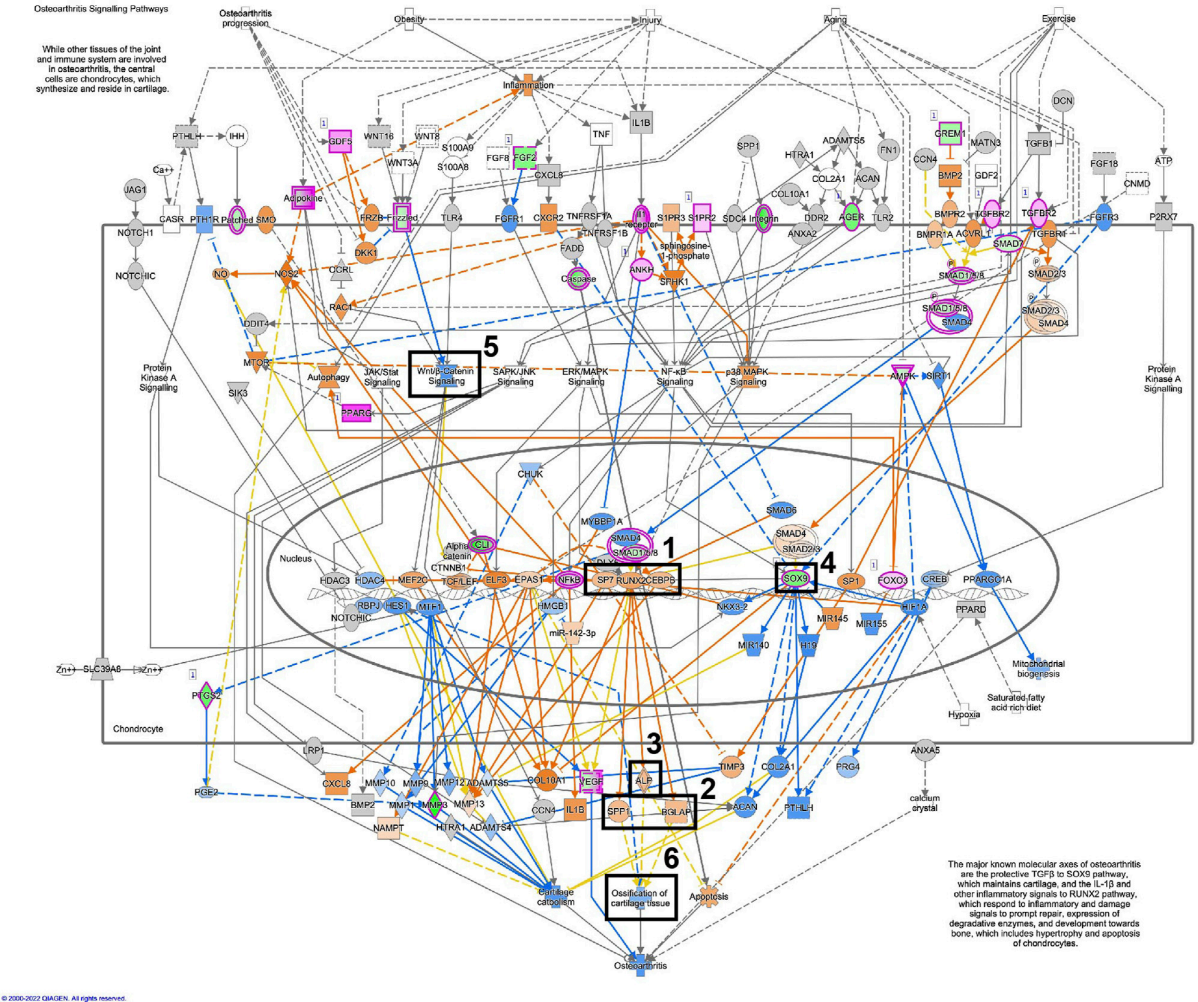
Osteoarthritis pathway is activated in osteoblastic differentiated fibroblasts. A detailed illustration of the osteoarthritis pathway in osteoblastic differentiated fibroblasts, based on the RNA-seq data. The most essential osteoblastic markers and processes have been highlighted in five boxes (1–5). Box 1 (SP7, RUNX2, CEBPB), Box 2 (SPP1/osteopontin, BGLAP/osteocalcin), Box 3 (ALP/alkaline phosphatase), Box 4 (Sox9), Box 5 (Wnt/β-catenin signaling), Box 6 (Ossification of cartilage tissue). For the pathway map, genes with an expression fold change of log2FC > abs (2) and p_adj. <0.01 were selected from the RNA-seq data. Genes colored in pink are up-regulated, and genes colored in green are down-regulated. Genes colored in orange are predicted to be up-regulated, and genes colored in blue are predicted to be down-regulated, based on measured expression values from the dataset.

**FIGURE 12:**
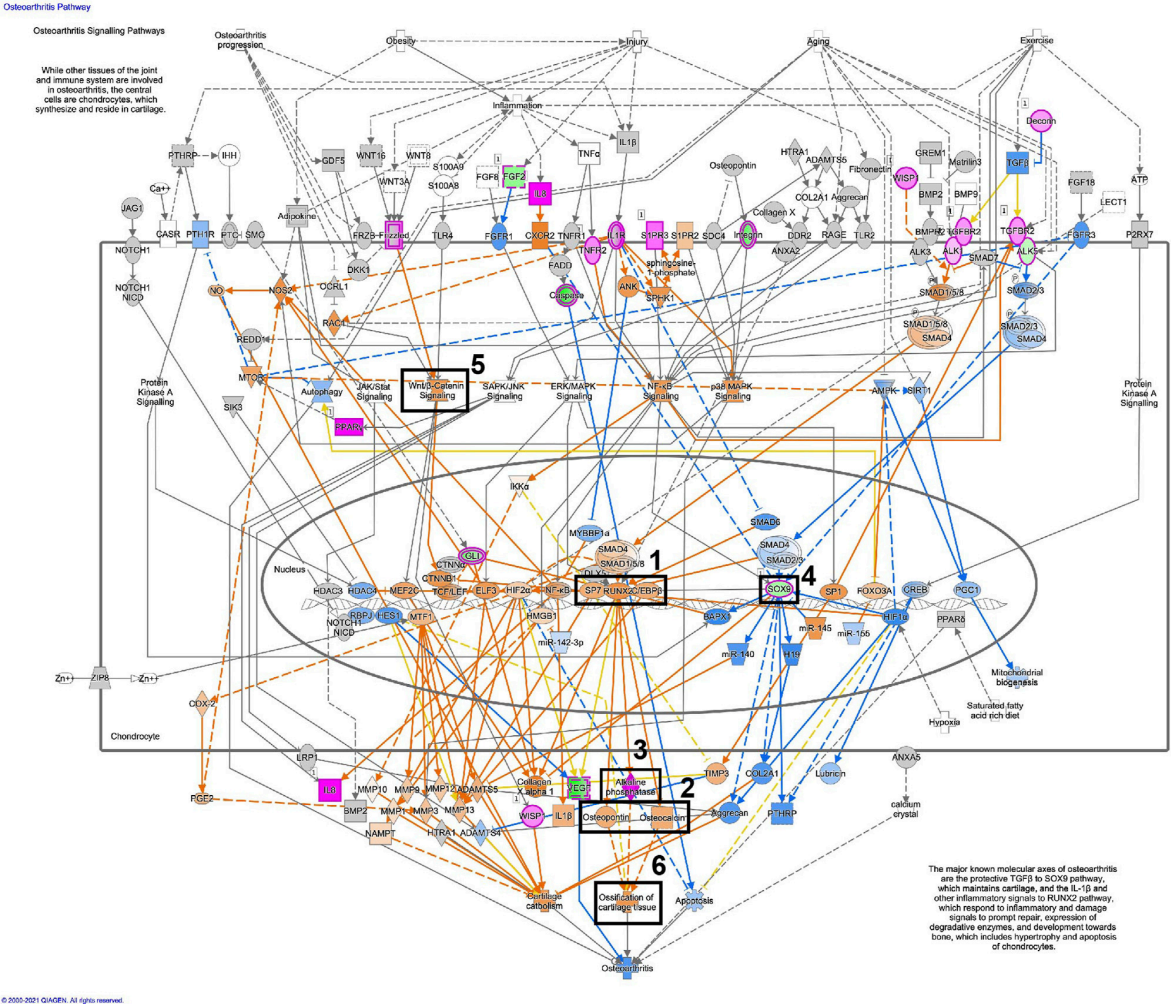
Osteoarthritis pathway is activated in osteoblastic differentiated MSCs. Detailed illustration of the osteoarthritis pathway in osteoblastic differentiated MSCs, based on the RNA-seq data. The most essential osteoblastic markers and processes have been highlighted in five boxes (1–5). Box 1 (SP7, RUNX2, CEBPB), Box 2 (SPP1/osteopontin, BGLAP/osteocalcin), Box 3 (ALP/alkaline phosphatase), Box 4 (Sox9), Box 5 (Wnt/β-catenin signaling), Box 6 (Ossification of cartilage tissue). For the pathway map, genes with an p_adj. <0.01 were selected from the RNA-seq data. Genes colored in pink are up-regulated, and genes colored in green are down-regulated. Genes colored in orange are predicted to be up-regulated, and genes colored in blue are predicted to be down-regulated, based on measured expression values from the dataset.

Figure 11, displays the osteoarthritis pathway in osteoblastic differentiated fibroblasts, based on the RNA-seq data. A similar illustration in osteoblastic differentiated MSCs is displayed in Figure 12. These analyses suggest activation of essential osteoblastic differentiation genes, including *SPP1*, *BGLAP*, *SP7*, *RUNX2*, and *CEBPB* (Figures 11, 12; box 1 and 2). In addition, the illustrations show a predicted activation of *ALPL* in fibroblasts and a predicted up-regulation of *ALPL* in MSCs (Figures 11, 12; box 3). Finally, *SOX9* is down-regulated in both cell types (Figures 11, 12; box 4). Some differences between the cell types are also seen. The important osteogenic WNT/β-catenin signaling has a predicted activation in MSCs but a predicted inhibition in fibroblasts, while ossification of cartilage tissue shows activation in MSCs and inhibition in fibroblasts (Figures 11, 12; box 5 and 6).

Together, these osteoarthritis pathway-related data further indicate that our *in vitro* treatment induces osteoblast-like differentiation in human fibroblasts that is comparable to the osteogenic differentiation in MSCs, and produces an *in vitro* osteoblastic system.

## Discussion

In this multi-omics study, we present a method and its in-depth validation for transdifferentiating human dermal fibroblasts into osteoblast-like cells. The *in vitro* treatment induced osteoblastic differentiation, demonstrated by high bone-specific ALP and deposited minerals, together with altered expression of central genes and proteins involved in the regulation of osteogenesis, as established by sequential transcriptomic, proteomic and phosphoproteomic profiling of transdifferentiating fibroblasts over a 35-day time course. The osteoblastic differentiation of human mesenchymal stem cells was investigated concurrently over a 28-day time course operating as a successful reference *in vitro* model system. Overall, we confirmed gene and protein expression profiles of the established osteogenesis markers, but also report new observations on factors whose association with bone formation is not so well known.

Skin biopsy collection from the forearm is a stress-free, minimally invasive procedure to harvest human dermal fibroblasts, which are easily maintainable and expandable *in vitro*. In addition, fibroblasts hold anti-inflammatory, immune modulatory and regenerative properties (Ichim et al., 2018). Hence, the dermal fibroblasts are considered as an excellent human source material for *in vitro* studies. In early 2000, Hee and Nicoll already proved that fibroblasts can be utilized in *in vitro* osteogenic studies, when they showed that dermal fibroblasts cultured in osteoblastic differentiation media were able to express osteoblastic markers (Hee and Nicoll, 2006). Here, our integrative study confirms their early findings related to fibroblasts’ osteogenic transdifferentiation, but on a far deeper level, incorporating multi-omics analyses to validate the results. Previous reports have also investigated the osteogenic potential of fibroblasts, however, the successful transdifferentiaton to osteoblast-like cells was performed either by adding an additional chemical component or induced virally, while in this study, the cells are exclusively cultured in osteoblastic differentiation media (Rutherford et al., 2002; Yamamoto et al., 2015; Yamamoto et al., 2018; Lu et al., 2020).

Using multi-omics approaches, the comprehensive view of all included datasets (RNA-seq, proteomics, phosphoproteomics) detected four distinct subgroups (untreated MSCs, untreated fibroblasts, differentiated MSCs and differentiated fibroblasts) and a high similarity in gene and protein expression profiles between the respective biological replicates in both cell types. Fibroblast 4, an outlier in the analysis, originated from a healthy individual with no known diseases or pathogenic mutations. On the other hand, the high similarity in gene and protein expressions between the fibroblast biological replicates still indicated that the profile of fibroblast 4 was similar enough to utilize as a replicate. The outlining might be due to a technical failure, perhaps in RNA-and protein isolation. Overall, the osteoblastic differentiation treatments induced both similar and different expression profiles in fibroblasts and MSCs. The RNA-seq data showed a bigger variation of differentially expressed genes between the two *in vitro* cell model systems, while the proteomic data expressed more similarities. The RNA-seq also contained, in total, more differentially expressed genes than the differentially expressed proteins in the proteomic dataset, which could explain this occurrence. In addition, all RNA alterations are not always mediated to protein level, e.g. due to posttranscriptional regulation. However, it is important to remember that the starting material differs in the two *in vitro* model systems, indicating that there may be differences in expression profiles of various genes, even if the expression of osteogenic markers is parallel.

During osteogenic differentiation, MSCs and fibroblasts are expected to lose their cell-type specific identity (Olsen et al., 1989; Balzar et al., 1999; Maleki et al., 2014; LeBleu and Neilson, 2020; Muhl et al., 2020). Indeed, our RNA-seq and proteomic data show that during the osteoblastic differentiation treatment, the expression of these specific markers plateaued simultaneously as the expression of essential osteoblastic markers peaked. Early osteoblastic differentiation includes down-regulation of *SOX9* and up-regulation of *RUNX2* and *ALPL* (Khurana et al., 2018). RUNX2 is essential for bone mineralization (Huang et al., 2007). Our *in vitro* technique induced a decrease in *SOX9* gene expression and elevated gene and protein expression of RUNX2 and ALPL in both cell types as the differentiation continued. Notably, COL1A1, with an elevated expression profile in fibroblasts, is also an early marker of osteoblast differentiation (Huang et al., 2007). During osteoblastic treatment, COL1A1 expression decreased in fibroblast but apparently remained sufficient to promote osteogenesis.

Importantly, other essential osteoblastogenesis genes such as different collagens, extracellular matrix genes and many osteogenesis genes including *SMADs*, *BMPs,* and *TGF-β* also altered accordingly. Following TGF-β/BMP induction during osteogenesis, both the SMADs and p38 MAPK pathways promote *RUNX2* expression to control osteoblastic differentiation of precursor cells. The coordinated activity of RUNX2 and TGF-β/BMP-activated SMADs is critical for formation of the skeleton (Chen et al., 2012). Up-regulated extracellular matrix genes in both cell models include *PHEX*, *MGP*, and *MMP13*. *PHEX* is expressed in bone and its inactivation triggers impaired mineralization (Liu et al., 2002). *MGP* expression increases during matrix development and *MMP13* is known to be highly expressed in differentiated osteoblasts (Barone et al., 1991; Siqueira et al., 2011). Other osteogenesis genes, such as *EGFR*, *PTH1R*, *BMP6*, and *IGF2* were also up-regulated. *EGFR* stimulates *EGR2* expression, which is critical for osteoprogenitor maintenance and new bone formation (Chandra et al., 2013). Mice with a conditional deletion of *Pth1r* in osteoblasts show disrupted trabecular bone formation (Qiu et al., 2015). *BMP6* strongly induces ALP expression and *MSX1* down-regulates cholesterol synthesis-related genes to ensure osteoblastic differentiation, since cholesterol inhibits osteoblastic differentiation (Ebisawa et al., 1999; Goto et al., 2016). *IGF1* is an osteoblast-stimulating factor and *IGF2* was found to promote ALP activity or collagen synthesis in differentiated osteoblasts (Hamidouche et al., 2010). Essential down-regulation of, for instance, *PLOD2*, *HOXA2,* and *ZNF384* was seen in both cell types during the osteoblastic differentiation. *PLOD2*, a collagen cross-link regulator, is suppressed in the late stage of osteoblast differentiation (Qi and Xu, 2018). *HOXA2* inhibition promotes osteogenic differentiation whereas *Hoxa2* ^*−/−*^ mouse embryonic palatal mesenchyme cells exhibited increased bone matrix deposition and mineralization *in vitro* (Iyyanar and Nazarali, 2017). Finally, *ZNF384* deficiency enhances BMPs and induces osteoblastic differentiation in bone marrow cells in cultures (Morinobu et al., 2005). In addition, to these up- and down-regulations, fibroblasts and MSCs stained positive for bALP, a pre-osteoblastic marker, after 14 days of treatment verifying the results of a partial osteogenic differentiation. Further, both cell types showed deposited calcium and phosphate at endpoint implying development of mature osteoblasts/osteoblast-like cells. Importantly, the RNA-seq data were validated using qRT-PCR, indicating that the presented RNA-seq data is reliable. For instance, elevated expression of *ALPL* and *TGFBR2*, two central osteogenic genes, can be detected in both the RNA-seq data and with qRT-PCR.

Utilizing transcriptomic, proteomic, and phosphoproteomic analyses, we were also able to identify novel factors related to osteogenesis. For instance, in the RNA-seq data, we detected an up-regulation of *SOD2*, an eliminator of oxidation in mitochondria, which plays an essential role in osteoblastic differentiation and bone formation by regulating mitochondrial stress (Gao et al., 2018). In addition, elevated mRNA expression of *CILP* was detected in fibroblasts and MSCs. CILP is known to induce mineralization when expression levels are elevated, and it has been reported that, together with *Phex*, the expression levels are markedly up-regulated in calvarial osteoblasts *in vitro* (Staines et al., 2014). Meanwhile, we also observed increased mRNA expressions of *JAK2* in both cell types. Inhibition of *JAK2* in bone marrow MSCs reduces ALP activity and matrix mineralization (Yu et al., 2018). Furthermore, genes linked to cholesterol metabolism, such as *NCEH1* and *LRP8*, and proliferation-associated gene *PDK1*, were down-regulated in both cell types during the treatment. These are important findings, since free cholesterol may inhibit BMP2 to block the expression of *RUNX2*, ALPL, and COL1A1 in osteoblast cells, which in turn inhibits osteoblastic differentiation, and during cell differentiation the proliferation gets stalled (Nakamura et al., 2008; Dolley et al., 2011; Ruijtenberg and van den Heuvel, 2016; Zhang et al., 2017; Yin et al., 2019). These factors might be considered new osteoblastic markers but more functional studies are needed to prove relevance for osteoblast differentiation.

The proteomic data showed only 293 and 130 significantly (p_adj. <0.05) differentially expressed proteins in differentiated fibroblasts and MSCs, respectively, at endpoint. Only a few of the differentially expressed proteins, coded by the essential osteogenesis-linked genes shown in Figure 9, were significantly expressed in both cell lines. Therefore, we focused on six other, not so well-known, proteins that can be associated with osteogenesis, including POSTN, S100A11, ANXA4, FBLN1, EFEMP2, and NANS. For instance, we detected increased protein (and mRNA) expression of FBLN1, which is needed for bone formation and Bmp-2-mediated stimulation of Osterix required for osteoblastic differentiation (Cooley et al., 2014; Hang Pham et al., 2017). Elevated protein expression of NANS and ESD was also detected. These proteins are involved in the synthesis and recycling of sialic acid, which in turn is needed for the expression of important bone mineralization factors such as of bone sialoprotein (BSP), osteoprotegerin (OPG), and vitamin D receptor (VDR) (Xu et al., 2013; van Karnebeek et al., 2016). In contrast to all the similarly regulated proteins, we also detected different up- and down-regulation profiles between the cell types. For instance, CDH2 is up-regulated in fibroblast but down-regulated in MSCs at treatment endpoint. During osteogenesis, CDH2 is upregulated but when the cells differentiate towards osteocytes, the expression starts to decrease (Marie et al., 2015). This may indicate that MSCs have differentiated further on the osteogenic lineage, than fibroblasts. Proteomic data analysis frequently encounters problems with missing values that greatly reduce the confidence of the data, which would justify the low rate of significant differentially expressed proteins (Jin et al., 2021). However, with an appropriate imputation method, in our case the MinProb (Probabilistic Minimum Imputation Method) with a single value imputation approach, the missing value can be replaced, and the whole dataset analyzed (Lazar et al., 2016). The authenticity of the analysis done on imputed values should be trusted, since this is the common approach in the field of proteomic analysis. We confronted similar issues with the phosphoproteomic analysis identifying very few significant differentially expressed phosphoproteins in differentiated MSCs and fibroblasts at endpoint. However, we observed one interesting finding, a hyperphosphorylation of PML on position S403 in both fibroblasts and MSCs. Overexpression of PML promotes up-regulation of bone sialoprotein, which inhibits cell proliferation and induces osteogenic differentiation (Sun et al., 2013). Nevertheless, the differentially expressed proteins and phosphoproteins presented in Figure 5 together with all obtained data in the study, are sufficient to suggest that the *in vitro* method promotes an osteoblast-like phenotype.

Other fundamental genes and pathways, besides from *RUNX2*, *SOX9,* and *ALPL*, that are vital for osteoblastogenesis, are Wnt-signaling, osterix (*SP7*), osteopontin (*SPP1*) and osteocalcin (*BGLP*) (Huang et al., 2007). Unfortunately, none of these factors passed the filtration in RNA sequencing analysis and no gene expressions could be detected. This could be attributable to the fact that low-expression genes may be indistinguishable from sampling noise during filtering (Sha et al., 2015). However, based on the IPA analysis, one of the most likely activated pathways was the osteoarthritic pathway. In this pathway, it was predicted that *SP7*, *SPP1,* and *BGLP* were activated or with a high possibility expressed. The activation of the osteoarthritic pathway itself is also an important finding since osteoblasts have an essential role in osteoarthritis. Osteoblasts are one source of cells that produce transcription factors and growth hormones that are involved in the pathogenesis of osteoarthritis (Maruotti et al., 2017). In addition, the pathway of atherosclerosis was highly predicted to be activated. This finding is also conclusive with a predictable osteoblastic phenotype since it has been shown that calcified atherosclerotic arteries can contain tissue that is histomorphologically indistinguishable from bone and that the process of calcifying atherosclerotic plaque is viewed as a process that exhibits similarities to bone formation (Doherty Terence et al., 2003).

Finally, we want to emphasize the important finding of the regulated gene expressions of gene sets associated with G-protein coupled receptors, extracellular matrix organization processes, and collagens, as well as with the complement cascade (part of the immune system) in differentiated MSCs and fibroblasts. There is a well-known association between collagens and extracellular matrix (ECM) organization processes and osteogenesis, since collagens are the most abundant component of the bone ECM. The ECM in turn is involved in regulating e.g. proliferation, differentiation and the functional characteristics of the mature bone (Lin et al., 2020). In addition, multiple human GPCR mutations impair bone development or metabolism (Luo et al., 2019). The most interesting finding was the link to the complement system. Complement components influence endochondral bone formation and affect the skeletal macroscopic structure and architecture (Mödinger et al., 2018). Furthermore, osteoblastic differentiation has been shown to involve the up-regulation of several complement proteins, supporting our results (Mödinger et al., 2018).

We recognize some limitations in our study. Our multi-omics data were based on only three commercial MSC lines and four human dermal fibroblast lines in the multi-omics data analysis. Technical replicates of the same samples would have been preferable, which indeed could have prevented the deviant fibroblast cell line in the PCA plot. However, we performed the histological experiments with several technical replicates and RNA validation and RNA-seq analysis utilized total RNA extracted from separate samples. Furthermore, it would have been beneficial to utilize cell models of non-connective tissue (epithelial, hematopoietic), as a negative reference, to show that the osteoblast-like response in fibroblasts is credible. In the study, MSCs were utilized as a positive control. An additional way to ensure osteoblastic phenotype would be to compare differentiated fibroblast at endpoint to osteoblast cells. However, such an approach was not possible since osteoblasts from healthy individuals were not available. Additional limitations are the challenges with the proteomic and phosphoproteomic data, which led to the detection of a limited number of differently expressed proteins and phosphoproteins modulated by the osteoblastic differentiation treatment. Finally, we mainly focused on the profile comparison between day 0 and treatment endpoint in both MSCs and fibroblast. However, in a follow-up study, other comparisons, such as day 0 *versus* day 14 or day 14 *versus* treatment endpoint, would be intriguing to perform to get more in depth knowledge on GO terms covering narrower time frames but at distinct moments during differentiation.

In conclusion, we present here an *in vitro* technique to transdifferentiate human dermal fibroblasts to osteoblast-like cells. With these data, we provide an in-depth insight to the developmental processes toward osteoblasts-like cells and elucidate new possible markers related to osteogenesis. This *in vitro* technique can be utilized as an alternative model to mesenchymal stem cells in modeling skeletal disorders, associated with impaired osteoblast function, to study underlying disease mechanisms. Future studies should explore the utility of this method in investigation of osteoblastic defects in patients with a genetic bone disease.

## Data Availability

The datasets presented in this study can be found in online repositories. The names of the repository/repositories and accession number(s) can be found below: https://a3s.fi/FBOB/Makitie_RNAseq_raw_count_matrix.xlsx, CSC-IT Center for Science, https://a3s.fi/FBOB/MSC_results_phospho_28012021.xlsx, CSC-IT Center for Science, https://a3s.fi/FBOB/MSC_results_total_28012021.xlsx, CSC-IT Center for Science, https://a3s.fi/FBOB/Phospho_results_fibroblast.xlsx, CSC-IT Center for Science, https://a3s.fi/FBOB/Results_total_fibroblast.xlsx, CSC-IT Center for Science.
