# Supplementary material for: A multi-omics study to characterize the transdifferentiation of human dermal fibroblasts to osteoblast-like cells
Source: Front Mol Biosci. 2022 Nov 17;9:1032026. doi: 10.3389/fmolb.2022.1032026 (PMC9714459; doi:10.3389/fmolb.2022.1032026)
Supplement: Supplementary file 2 [file DataSheet2.docx]

Supplement 2


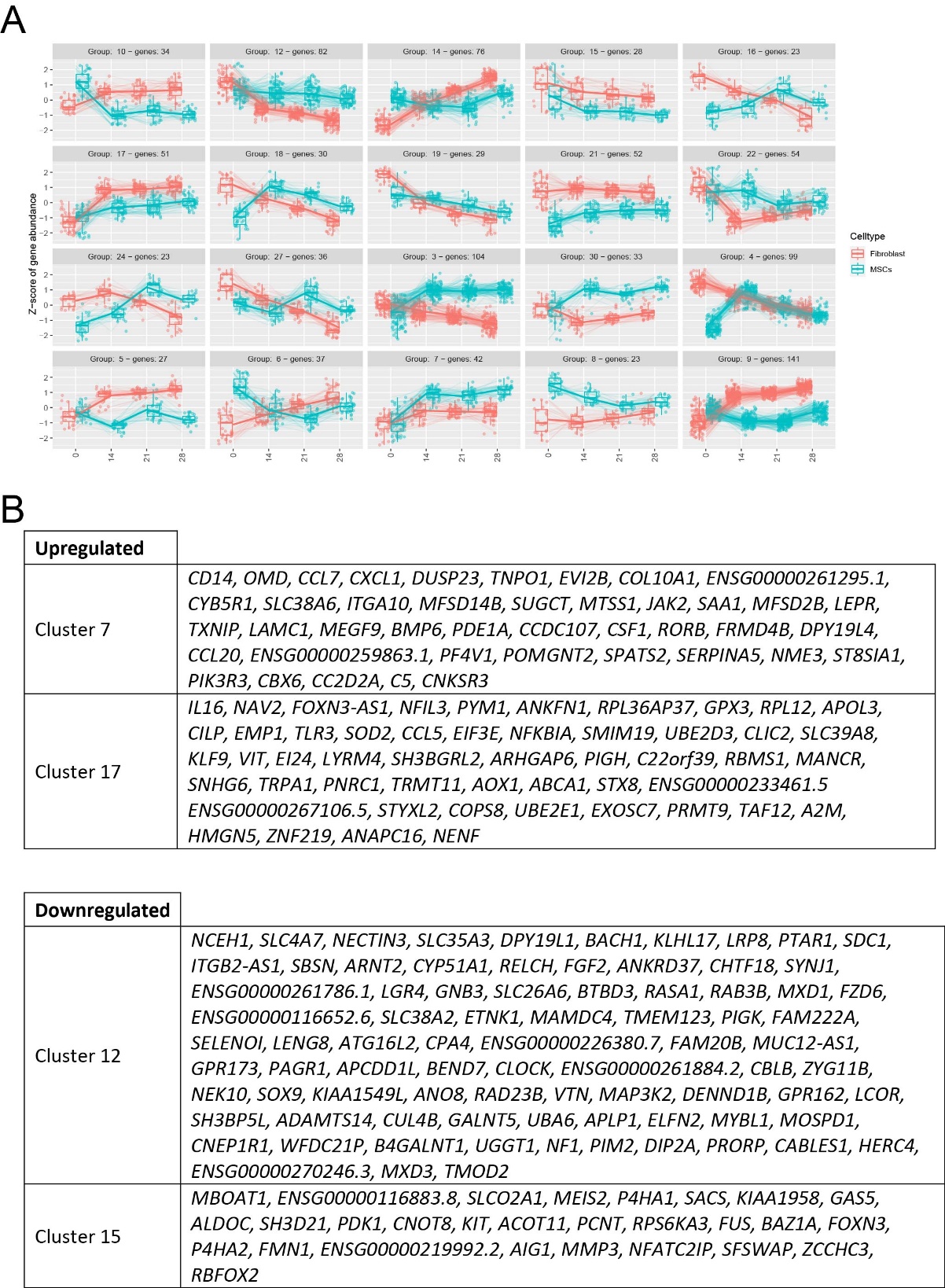


**Suppl. Figure 1. Similar gene cluster profiles expressed in MSCs and fibroblast during the treatment.** (A) A total of 1,024 genes, listed in Sl. 3E, were distributed across 20 clusters of various sizes visualizing both up- and down-regulated genes in fibroblasts and MSCs during the osteoblastic treatment. (B) Group 7 and 17 show upregulated genes in both cell types during the treatment while group 12 and 15 showed clusters of genes with a decreased expression. Cluster 7 consisted of 42 genes, cluster 17 of 51 genes, cluster 12 of 82 genes and cluster 15 of 28 genes. For clustering, all genes that were significantly differently expressed with a p_adj. <0.05 in any of the time points from the course analysis were taken as an input.


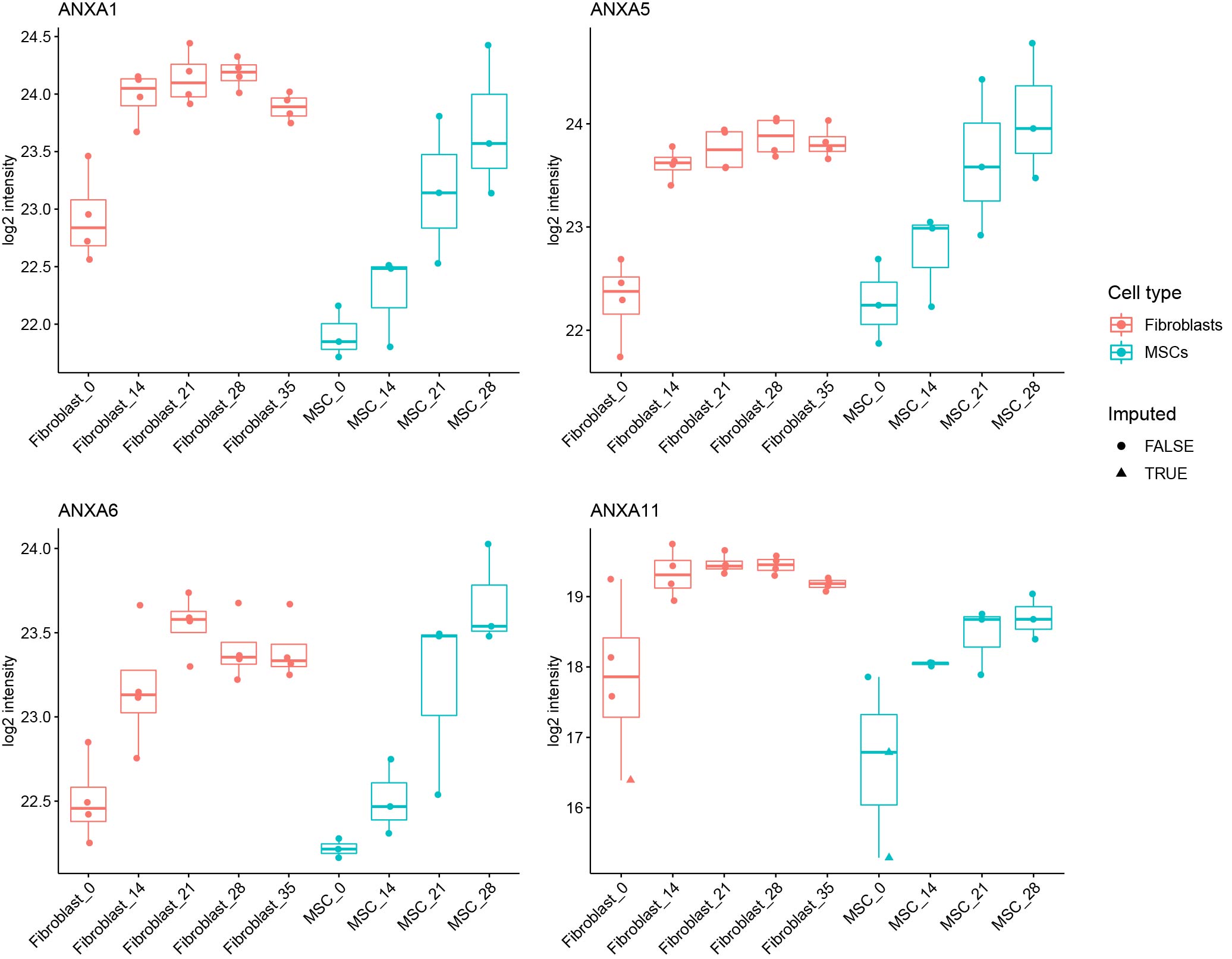


**Suppl. Figure 2. Protein expression Annexins in osteoblastic differentiation.** ANXA1, ANXA5, ANXA6 and ANXA11 showed elevated protein expression in fibroblasts and MSCs during the osteoblastic treatment. Box plots showing the VSN-normalized protein intensities of each of the differently expressed proteins across all fibroblast and MSC samples were generated, indicating imputed and non-imputed values with different symbols. P_adj. <0.05(Benjamini-Hochberg FDR adjustment).


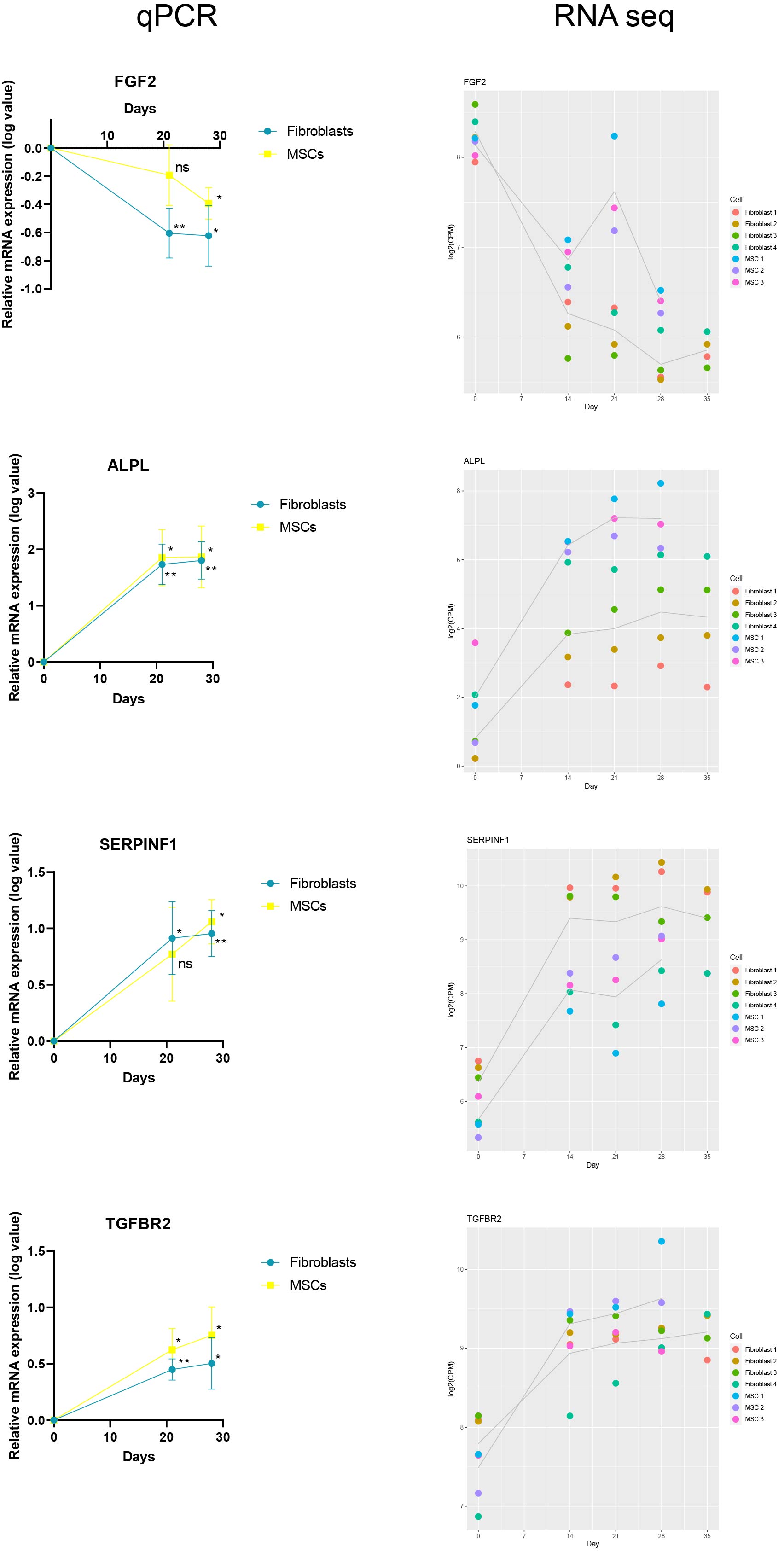


**Suppl. Figure 3. RNA-seq validation implying trustworthy results.** (A) Validation of RNA-seq data by detecting the relative mRNA expression of *FGF2*, *TGFBR2*, *ALPL* and *SERPINF1* using qPCR. The gene expressions were normalized to TBP according to the ΔΔ Ct method. Similar gene expression profiles of these target genes are distinguished in qPCR and with RNA-seq. Significant expression with qPCR were analyzed with paired t-test, two-tail (ns > 0.05, * < 0.05, ** < 0.01), mean ± SD. Gray lines represent cell-type-specific average expression levels. For mRNA expression plots, log2-transformed counts per million (CPM) values were used.


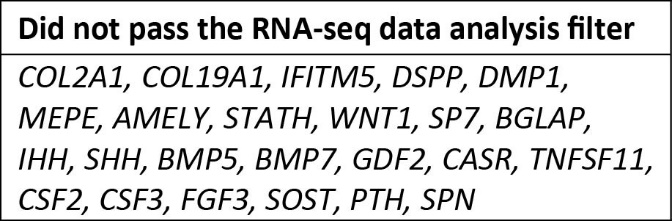


**Suppl. Figure 4. Essential genes of osteoblastogenesis that failed the RNA-seq criteria.** Twenty-four fundamental bone associated genes were not included in the RNA-seq differential expression analysis as their expression was not measured at detectable levels, here CPM > 0.12 in at least 4 samples (Median library size is 81 mio; we want to throw genes with less than 10 raw counts in 4 samples -> 10/81 = 0,12 -> the filterByExpr edgeR function keeps genes that have a CPM of 0,12 or more in at least four samples).


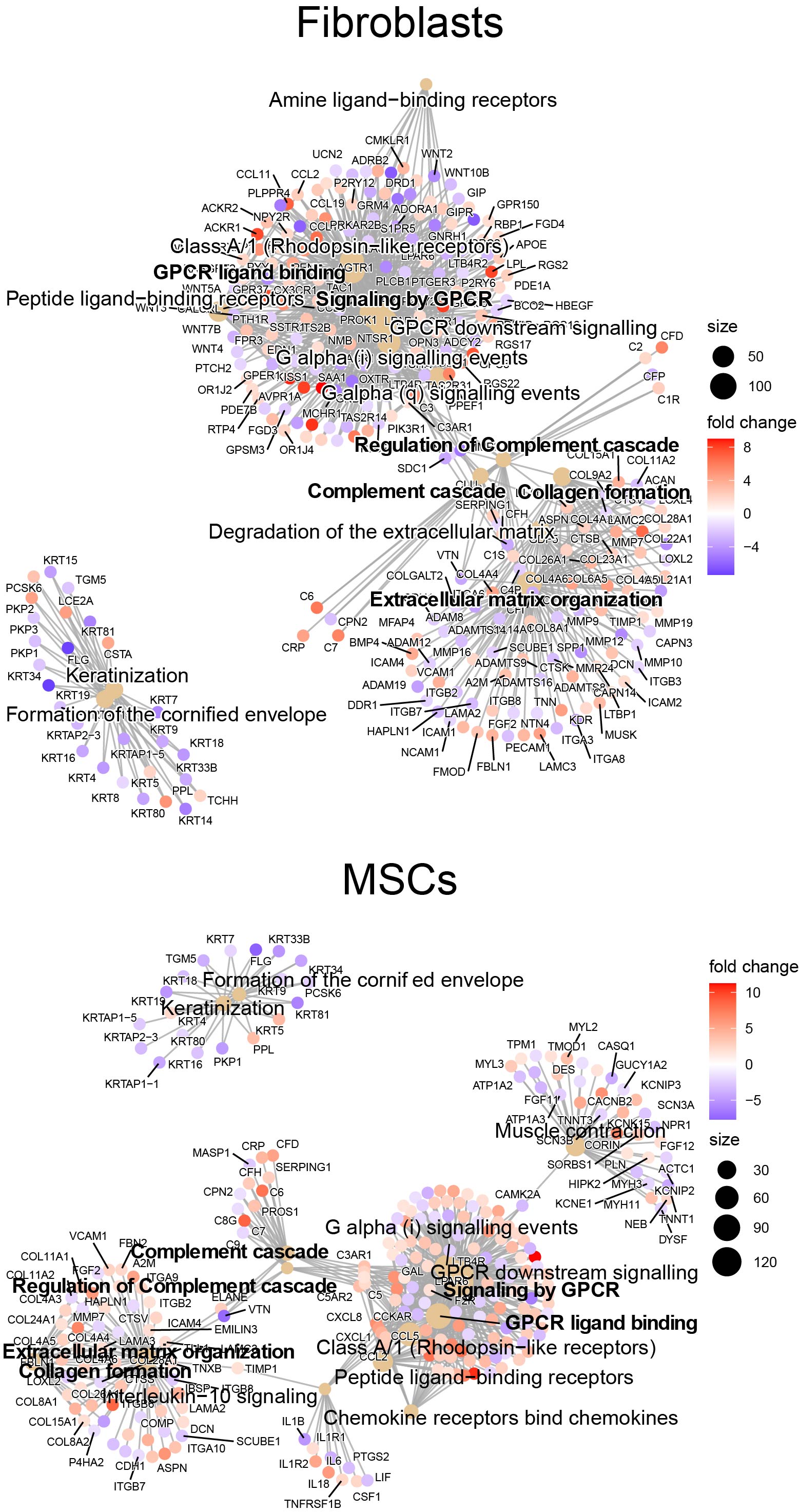


**Suppl. Figure 5. Overrepresentation analysis (ORA) of differently expressed genes, from baseline to treatment endpoint, in MSCs and fibroblast.** The top 15 most significant gene sets (Reactome pathways) are illustrated in more detail. The most essential pathways are bolded. All significant genes with log2FC > abs(1.5) have been visualized. The up-regulated genes are colored in shades of red, and down-regulated genes in shades of blue. The size of the central network node reflects the number of genes in the pathway. Analysis was performed on differentially expressed genes of MSC 28 Day and Fibroblast 35 Day. Differentially expressed genes for the enrichment analysis were chosen using adjusted p-value threshold of 0.05 and requiring at least 1.5-fold up- or down-regulation in expression.
