# Supplementary material for: A multi-omics study to characterize the transdifferentiation of human dermal fibroblasts to osteoblast-like cells
Source: Front Mol Biosci. 2022 Nov 17;9:1032026. doi: 10.3389/fmolb.2022.1032026 (PMC9714459; doi:10.3389/fmolb.2022.1032026)
Supplement: Supplementary file 3 [file DataSheet1.docx]

Supplement 1

Differentiation protocol

**Table 1. Differentiation protocol of fibroblasts**

|  | **Concentration** | **Day 0-7** | **Day 7-35** | **Product** |
| --- | --- | --- | --- | --- |
| Advanced MEM (minimum essential media) | - | x | x | *12492013, Gibco* |
| Glutamax | 1 % | x | x | *35050-038, Gibco* |
| Penicillin/streptomycin | 1 % | x | x | *15140122, Gibco* |
| FBS | 10 % | x | x | *S-FEB-SA-015*  *Serana* |
| L-ascorbic acid | 0.3 mM | x | x | *A4544-25g, Sigma* |
| β-glycerophosphate | 10 mM | x | x | *G9422-50g, Sigma* |
| Dexamethasone | 100 nM | x |  | *D8893-1MG, Sigma Aldrich* |
|  | 10 nM |  | x |  |

**Table 2. Differentiation protocol of MSCs**

|  | **Concentration** | **Day 1-7** | **Day 7-28** | **Product** |
| --- | --- | --- | --- | --- |
| α-MEM ( alpha-minimum essential media)  - L-glutamine  - ribonucleosides  -deoxyribonucleosides | - | x | x | *41061029, Gibco* |
| Penicillin/streptomycin | 1 % | x | x | 15140122, Gibco |
| FBS | 10 % | x | x | 160000044, Thermo Fisher |
| L-ascorbic acid | 0.05 mM | x | x | A4544-25g, Sigma |
| β-glycerophosphate | 10 mM | x | x | G9422-50g, Sigma |
| Dexamethasone | 100 nM | x |  | D8893-1MG, Sigma Aldrich |
|  | 10 nM |  | x |  |

**RNA seq**

Alignment and gene counting

The RNA-seq libraries were generated with the Illumina TruSeq Stranded mRNA kit, as strand-specific poly(A)-selected libraries, sequenced to 150-bp paired-end reads. Total sample was 32. Samples were sequenced on NovaSeq6000 (NovaSeq Control Software 1.7.5/RTA v3.4.4) with a 151nt(Read1) 10nt(Index1)-10nt(Index2)-151nt(Read2) setup using 'NovaSeqXp' workflow in 'S4' mode flowcell. The Bcl to FastQ conversion was performed using bcl2fastq_v2.20.0.422 from the CASAVA software suite. The quality scale used is Sanger / phred33 / Illumina 1.8+.

Trimming was performed with Cutadapt [Martin, 2011]. Trimmed RNA-seq reads were aligned to the human reference genome GRCh38 using STAR [Dobin et al., 2013]. Duplicate-marked and sorted aligned bam files were used gene counting. Gene-level read counts were obtained using htseq-count [Putri et al., 2021] with union mode and --stranded reverse parameter, with transcriptome annotation GTF file version GRCh38.99.

Expression levels of marker genes

Normalized CPM values were computed by first using function calcNormFactors for calculating TMM-normalized (trimmed mean of M-values method) library sizes and then using cpm function of edgeR. The computed normalized CPM values were then log2-transformed, adding pseudo count of 1 to avoid negative values. Normalized Day 0 expression values were extracted for each two sets of marker genes. The values were visualised as barplots using ggbarplot function of R package ggpubr.

Differential expression analysis

All downstream analysis of the read count data was performed using R, version 3.6.1 [R Core Team, 2013]. MSC and Fibroblast samples were kept in the same data matrix, normalized and filtered together to have a unified gene set for the comparison of the MSC and Fibroblast differentiation process. The dataset was filtered with the edgeR function filterByExpr, leaving 18.469 genes for downstream analysis. The data was normalized using the TMM method with R package edgeR, version 3.32.1 [Robinson et al., 2010]. For visual exploration of the data, log2(CPM) values were used. Samples were visually inspected using Principal Component Analysis (PCA) and heatmaps of Pearson’s correlation distances using functions in the R base package stats. Differential expression (DE) analysis was conducted using the R packages *DESeq2*, version 3.32.1 [Love et al., 2014] and *limma*, version 3.46.0 [Ritchie et al., 2015]. For each time point, MSC samples or Fibroblast samples from that time point were joined and consider as biological replicates resulting in 3 replicates per time point for MSCs and 4 replicates per time point for fibroblasts.

Three types of analyses were performed: 1) MSC samples separately in end-point analysis with limma, comparing the last differentiation time point (28 days) to undifferentiated time point (0 days) using time as a factor, 2) Fibroblast samples separately in end-point analysis with limma, comparing the last differentiation time point (35 days) to undifferentiated time point (0 days) using time as a factor, 3) MSC and Fibroblast samples time course analysis in DESeq2 with likelihood ratio testing (LRT).

Limma evaluates Differential Expression (DE) by fitting gene-wise linear models to gene expression data, for each coefficient in the linear model or contrast, empirical Bayes (eBayes) moderated t-statistics and their associated P-values are used to assess the significance of the observed expression changes. P-values were then adjusted for multiple testing using Benjamini-Hochberg procedure [Benjamini & Hochberg, 1995]. The obtained results were then post-hoc filtered using 0.05 as the threshold for adjusted p-value (adj.pVal) reducing the probability of false discovery rate (FDR) and 1 as the threshold for absolute log2 fold change. Gene lists were extracted and genes were annotated with Ensemble IDs and human gene symbols using biomaRt, version 2.40.5 [Durinck et al., 2009].

DESeq2 provides a likelihood ratio test (LRT) when evaluating expression change across more than two levels, such as time in a differentiation time course experiment. Such a test aims to explore whether there are any significant differences in treatment effect between any of the timepoints or treatment types. For this test, two models are estimated per gene; the fit of one model is compared to the fit of the other model. DESeq2 implements the LRT by using an Analysis of Deviance (ANODEV) to compare the two model fits and calculate a pValue which are then adjusted for multiple testing using Benjamini-Hochberg procedure. For clustering, all genes that were significantly differentially expressed with an adj.p.Val < 0.05 in any condition from the time course analysis in DESeq2 were taken as input (1222 genes in total). The function degPatterns from the R package DEGreport was then used to identify gene clusters across the two different cell types. Regularised log-transformed (rlog) normalised gene expression counts were used. The degPatterns tool uses a hierarchical clustering approach based on pairwise correlations, then cuts the hierarchical tree to generate groups of genes with similar expression profiles. The tool cuts the tree in a way to optimise the diversity of the clusters, such that the variability inter-cluster > the variability intracluster.

Heatmaps of top 100 differential genes by significance were generated using R package pheatmap, v.1.0.12 [Kolde, 2018] using the normalised expression values of each sample and replicate. The log2 ratios were then used for generating clustered heatmaps in which genes and samples were hierarchically clustered using euclidean distances as the distance metric. In all of the heatmaps, data was scaled rowwise for better visualisation.

Overrepresentation analysis

Reactome pathway over-representation analyses (ORA) were performed using R package clusterProfiler [Yu et al., 2012]. The analyses determined whether any terms are annotated to a list of specified genes, in this case a list of differentially expressed genes, at a frequency greater than what would be expected by chance, and calculated a p-value using the hypergeometric distribution. The total set of genes from the dataset was used as a list for background genes. This analysis was performed on differentially expressed genes of MSC 28 Day DEGs and Fibroblast 35 Day DEGs. Differentially expressed genes for the enrichment analysis were chosen using adjusted pValue threshold of 0.05 and requiring at least 1.5-fold up- or down-regulation in expression.

The minimum and maximum gene set sizes were set to 10 and 500, respectively. Resulting p-values were adjusted using the Benjamini-Hochberg procedure. Enriched terms were further visualized using clusterProfiler functions (dotplot, cnetplot)

Ingenuity pathway analysis

The Fibroblast endpoint results (dataset 1) and MSC endpoint results (dataset 2) were analysed with Ingenuity pathway analysis (IPA). Differentially expressed genes were defined using the following cut-offs: a) adjusted p-value < 0.01, b) absolute log2 fold change > 1. Resulting DEG lists were 1217 genes and 726 genes for datasets 1 and 2, respectively. All analysed genes in the respective datasets were used as the background genes for control. IPA Core Analysis results for Canonical Pathways, Upstream Regulators, Master Regulators and Regulator Effects were exported as Excel tables, adjusted p-value 0.05 was used as a threshold for filtering for statistically significant results.

**Total proteomics**

Database Search and Quantitation

Raw data were processed with MaxQuant version 1.6.0.16 [Cox & Mann, 2008]. MS spectra were compared against the human component of the UniProtKB database (release 2020_01 with 20,303 entries) using the Andromeda search engine. Carbamidomethylation (+57.021 Da) of cysteine residues was used as static modification. Phosphorylation of serine/threonine/tyrosine (+79.966 Da) and oxidation (+15.994 Da) of methionine were used as dynamic modification. Precursor mass tolerance and fragment mass tolerance were set to less than 20 ppm and 0.1 Da, respectively. A maximum of two missed cleavages was allowed. The results were filtered to a maximum false discovery rate (FDR) of 5 %, and phosphosamples further based on the phosphorylation localization probability at the cut-off value of 0.75 with exception of two adjacent sites where the cut-off value was 0.5.

Data, quality control and pre-processing

The data was delivered as raw LFQ intensity values from an LC-MS/MS analysis. Data from MSC and fibroblasts were combined, totaling to 3091 proteins. R package DEP v. 1.14.0 [Zhang et al., 2018] was used for subsequent data normalization, filtering and imputation steps.

Basic filtering of DEP package was applied, filtering for proteins that were identified in all replicates in at least one condition (condition = cell type + time point). 1235 proteins were retained for subsequent analysis. Normalization of the data was done using variance stabilizing normalisation (VSN) that addresses the dependence of the variance on the mean intensity. Box plots of the data before and after normalization were generated to inspect the result of the normalization procedure. DEP plotting functions were used for plotting a heatmap that showed the missing value pattern as well as a barplot showing numbers of observed proteins per sample after the filtering. Data imputation was performed using the MinProb method (using probabilistic minimum values). Principal Component Analysis (PCA) was performed using plot_pca function of DEP for non-imputed and imputed data using the top 300 most variable proteins to inspect the affect of the data imputation on the sample grouping.

Endpoint differential expression analysis

Differential expression (DE) analysis based on linear models and empirical Bayes statistics was implemented using the function test_dif in the R package DEP that is calling linear models from R package limma. The two sample sets, MSC samples and primary fibroblasts, were analysed by contrasting the last time point (day 28 or day 35 for MSC and fibroblasts, respectively) to the day 0 time point. Significantly changed proteins were marked by defining 0.05 as the threshold for the adjusted pvalue (Benjamini-Hochberg). K-means (k = 6) clustered heatmaps of the log2 intensities of all significant proteins with the data centered per protein were generated as well as volcano plots showing the log2 fold changes on x-axis and the corresponding adjusted p-values in y axis. Box plots showing the VSN-normalised protein intensities of each of the DE proteins across all fibroblast and MSC samples were generated, indicating imputed values from non-imputed values with different point shapes (0.05).

Ingenuity pathway analysis

Both the MSC and primary fibroblast data were subjected to the Core Analysis of the Ingenuity Pathway Analysis (IPA) software. The DEPs to be analysed were defined using a significance cutoff of adj.p.Val < 0.05 and biological significance cutoff abs(FC) > 2. All measured proteins in the respective datasets were used as the background proteins. IPA Core Analysis results for Canonical Pathway enrichment, Upstream Regulators, Master Regulators and Regulator Effects were exported as Excel tables. Adjusted p-value 0.05 was used as a threshold for filtering for statistically significant results, if applicable. IPA Comparison Analysis tool was used for comparing the Core Analysis results of the MSC and fibroblast data. Heatmaps visualising the z-scores of the Canonical Pathway and Upstream Regulator results were generated, showing the results with p-value < 0.05 and absolute z-score > 2 in at least one of the datasets.

**Phosphoproteomics**

Database Search and Quantitation

As described in total proteomics.

Data, quality control and pre-processing

The data was delivered as raw intensity values from an LC-MS/MS analysis. R package PhosR v.1.2.0 [Yang et al., 2021] was used for constructing a PhosphoExperiment object using both MSC and fibroblast data, containing total of 4827 phosphosites in 1798 unique proteins. Unique phosphorylation site annotations were generated by combining UniProt accession, gene symbol, residue and site information. Data was then filtered using selectGrps function, keeping those phosphosites that have quantification of at least 50% of replicates in at least three conditions. 1806 phosphosites in 903 unique proteins were retained. Data analysis was continued using R package DEP v. 1.14.0 [Zhang et al., 2018] as the initial attempts to impute data using the PhosR resulted in dubious results.

Data normalization and data imputation were performed using DEP package as described for total proteomics, except that the data imputation method chosen for this data was k-nearest neighbour (knn) method. Before selecting the final imputation method, several methods, including two mixed imputation methods, were experimented (details not shown here). PCA plots for combined data as well as separate plots for MSC and fibroblast data were generated as described for total proteomics.

Endpoint differential expression analysis

Endpoint differential expression (DE) analysis of the phosphoproteins in MSC cells and primary fibroblasts were analyzed similarly as described for total proteins. Significantly changed phosphoproteins were marked by defining 0.05 as the threshold for the adjusted p-value (Benjamini-Hochberg).

References

Benjamini, Y., Hochberg, Y. (1995). Controlling the false discovery rate: a practical and powerful approach to multiple testing. J. R. Stat. Soc. B. 57, 289-300.

Cox, J., Mann, M. (2008). MaxQuant enables high peptide identification rates, individualized p.p.b.-range mass accuracies and proteome-wide protein quantification. Nat. Biotechnol. 26:12. doi: 10.1038/nbt.1511

Dobin, A., Davis, C.A., Schlesinger, F., Drenkow, J., Zaleski, C., Jha, S., et al. (2013). STAR: ultrafast universal RNA-seq aligner. Bioinformatics. 29. doi: 10.1093/bioinformatics/bts635

Durinck, S., Spellman, P.T., Birney, E., Huber, W. (2009). Mapping identifiers for the integration of genomic datasets with the R/Bioconductor package biomaRt. Nat. Protoc. 4:8. doi: 10.1038/nprot.2009.97

Kolde, R. (2018). Pheatmap: Pretty Heatmaps. R package version 1.0.10. https://CRAN.R-project.org/ package=pheatmap. [Accessed June 21, 2022]

Love, M.I., Huber, W., Anders, S. (2014). Moderated estimation of fold change and dispersion for RNA-seq data with DESeq2. Genome Biol. 15:12. doi: 10.1186/s13059-014-0550-8

Martin, M. (2011). Cutadapt removes adapter sequences from high-throughput sequencing reads. EMBnet.J. 17. doi:10.14806/ej.17.1.200

Putri, G., Anders, S., Pyl, P.T., Pimanda, J.E., Zanini, F. (2021). Analysing high-throughput sequencing data with HTSeq 2.0. arXiv:2112.00939. doi:10.48550/arXiv.2112.00939

R Core Team. (2013). R: A language and environment for statistical computing. R Foundation for Statistical Computing, Vienna, Austria. http://www.R-project.org/. [Accessed June 21, 2022].

Ritchie, M.E., Phipson, B., Wu, D., Hu, Y., Law, C.W., Shi, W., et al. (2015). limma powers differential expression analyses for RNA-sequencing and microarray studies. Nucleic Acids Res. 43:7. doi: 10.1093/nar/gkv007

Robinson, M.D., McCarthy, D.J., Smyth, G.K. (2010). edgeR: a Bioconductor package for differential expression analysis of digital gene expression data. Bioinformatics. 26:1. doi: 10.1093/bioinformatics/btp616

Yang, P., Kim, T., Kim, H.J. (2021). PhosR: A set of methods and tools for comprehensive analysis of phosphoproteomics data. R package version 1.2.0. https://github.com/PYangLab/PhosR. [Accessed June 21, 2022].

Yu, G., Wang, L.-G., Han, Y., He, Q.-Y. (2012). clusterProfiler: an R package for comparing biological themes among gene clusters. OMICS. 16:5. doi: 10.1089/omi.2011.0118

Zhang, X., Smits, A., van Tilburg, G., Ovaa H, Huber W, Vermeulen M. (2018). Proteome-wide identification of ubiquitin interactions using UbIA-MS. Nat. Protoc. 13:3. doi: 10.1038/nprot.2017.147
